# Enhancing the trustworthiness of chaos and synchronization of chaotic satellite model: a practice of discrete fractional-order approaches

**DOI:** 10.1038/s41598-024-60268-3

**Published:** 2024-05-09

**Authors:** Saima Rashid, Sher Zaman Hamidi, Saima Akram, Moataz Alosaimi, Yu-Ming Chu

**Affiliations:** 1 Department of Mathematics, Government College University, Faisalabad, 38000 Pakistan; 2 Department of Computer Science and Mathematics, Lebanese American University, Beirut, 11022801 Lebanon; 3 Department of Physics, Nangarhar University, Jalalabad City, Nangarhar 2601 Afghanistan; 4 Department of Mathematics, Govt. College Women University, Faisalabad, 38000 Pakistan; 5 Centre for Advanced Studies in Pure and Applied Mathematics, Bahauddin Zakariya University, Multan, 60000 Pakistan; 6 Department of Mathematics and Statistics, College of Science, Taif University, P.O. Box 11099, Taif, 21944 Saudi Arabia; 7 School of Science, Hunan City University, Yiyang, 413000 People’s Republic of China

**Keywords:** Fractional calculus, Satellite model, Fractional difference equation, Chaotic attractors, Bifurcation, Sample entropy, Lyapunov exponent, Space physics, Engineering, Mathematics and computing, Physics

## Abstract

Accurate development of satellite maneuvers necessitates a broad orbital dynamical system and efficient nonlinear control techniques. For achieving the intended formation, a framework of a discrete fractional difference satellite model is constructed by the use of commensurate and non-commensurate orders for the control and synchronization of fractional-order chaotic satellite system. The efficacy of the suggested framework is evaluated employing a numerical simulation of the concerning dynamic systems of motion while taking into account multiple considerations such as Lyapunov exponent research, phase images and bifurcation schematics. With the aid of discrete nabla operators, we monitor the qualitative behavioural patterns of satellite systems in order to provide justification for the structure’s chaos. We acquire the fixed points of the proposed trajectory. At each fixed point, we calculate the eigenvalue of the satellite system’s Jacobian matrix and check for zones of instability. The outcomes exhibit a wide range of multifaceted behaviours resulting from the interaction with various fractional-orders in the offered system. Additionally, the sample entropy evaluation is employed in the research to determine complexities and endorse the existence of chaos. To maintain stability and synchronize the system, nonlinear controllers are additionally provided. The study highlights the technique’s vulnerability to fractional-order factors, resulting in exclusive, changing trends and equilibrium frameworks. Because of its diverse and convoluted behaviour, the satellite chaotic model is an intriguing and crucial subject for research.

## Introduction

Chaotic systems are extremely responsive to initial conditions (ICs). The phenomenon is frequently referred to as the butterfly influence^1^. Chaos synchronization has garnered a lot of consideration in scientific circles since Pecora and Carroll^2^ developed the notion of chaotic synchronization under various ICs. The concept behind synchronization is to take advantage of the data generated by the centralized system in order to regulate the slave mechanism and guarantee its results adhere to the production of the acquire mechanism asymptotically^3,4^. One of the most crucial uses of chaos is the synchronization of multiple chaotic dynamical structures. Over recent decades, chaotic synchronization has emerged as an intriguing topic within the arena of scientific discipline owing to its broad range of conceivable uses in^5^. For tackling the synchronization of alike or non-similar master-slave chaotic structures, many techniques are being developed, including active supervision techniques, adaptable control approaches, fuzzy oversight procedures, back-stepping design techniques, impulsively regulation approaches, automatic control processes, parametric feedback controlling techniques and many more^6,7^.

Whenever the settings are unidentified or change over time, adaptive synchronization is used to synchronize the same or nonidentical mechanisms. Wang et al.^8^ investigated responsive synchronization for a Chen chaotic structure via entirely unresolved factors. Lin et al.^9^ contemplated the dynamic, powerful observer-based synchronization of unilaterally complemented chaotic networks with an unidentified transmit time delay. Fan et al.^10^ presented synchronization of a family of chaotic systems based on adaptive control design of input-to-state stability. Chen et al.^11^ expounded the hidden extreme multi-stability and synchronicity of memristor-coupled non-autonomous memristive Fitzhugh-Nagumo models.

With the advancement in space technology, the necessity of understanding satellite dynamics is of key importance for the improvement of satellite systems^5,6^. This system provides multiple positive effects over an individual rocket task, involving the capacity to boost and/or facilitate outreach by means of deeper starting point assessments, a high rate of failure tolerance, real-time reconfigurability, adaptability to extremely fluctuating requirements and lesser lifetime expenses^12^. Nevertheless, from spacecraft formation setup to transformation, interaction and pattern generation, the entire process presents enormous obstacles^13^. A single satellite is sometimes insufficient to accomplish certain missions of space observations and earth observations. These tasks can be accomplished using satellites synchronization. Replacing a single satellite with a number of smaller satellites in clusters helps to reduce the launching cost as well as reduces the risk of failure of a difficult and complex mission. Therefore, reduction in the launching cost and reduction in the risk of the entire mission failure are the other benefits of satellites synchronization^14–16^. Adaptive synchronization of a chaotic satellite attitude in the presence of external disturbances and uncertainties is difficult. Disturbances and uncertainties of the satellite attitude system are represented using auxiliary torques. The external disturbances are sunlight pressure torques, gravity gradient torques, aerodynamics moment, etc., whereas internal disturbances are model uncertainties and parametric uncertainties^17^. To address these issues, novel techniques for accomplishing satellite creation constellations while minimizing location maintenance necessities are being requested. The manoeuvring of satellites in their navigation is critical to security forces, courteousness and research endeavours. Satellite framework synchronization is an active academic field^18,19^. Several methods and procedures were implemented to synchronize and regulate nonlinear phenomena (satellite attitude), specifically responsive oversight, proactive surveillance and control using sliding mode^20,21^. The adaptable synchronization of satellite behaviour and momentum-based systems are complicated topics. The satellite behaviour framework includes unpredictability and disruptions (both exterior and interior). It’s a redundant torque structure. The disruptions in the outermost layer may encompass streamlined experiences, ultraviolet ray-compelled tensions, gravitation gradient forces, and electromagnetic instances, whereas internal fluctuations can embrace parameter unpredictability^22–24^. Satellite mechanism synchronization is currently employed in contemporary space-purpose theories featuring multiple satellites constellations with details. This is addressed by the synchronization regulation system, which regulates the variation in oversights within satellite constellations. The objective is to determine the advanced version of adaptive synchronization that motivates satellite constellations asynchronously regarding identical briefings^25,26^.

In the last century, discrete fractional (DF) calculus has grown up as an appealing study field that has sparked the fascination of researchers from multiple fields^27–31^. Their uses range from biological science to environmental science to practical scientific fields, providing useful understanding of contemporary issues^29,32–35^. In contrast to classical non-fractional networks, fractional platforms have proven their capacity to specify multifaceted chaotic events with more precision^36,37^. It highlights their distinctive features, such as persistent memory, transparency and adaptability. There is currently an increase in the number of articles presented on this fascinating subject^38–40^. Numerous researchers have proposed innovative formulations of discrete-time fractional calculus that have stability properties and multiple empirical results^41,42^. In particular, Wu and Baleanu^43^ offered groundbreaking research that explored the chaotic properties of fractional chaos illustrations employing the Caputo-type operator. As a consequence, this research has opened pathways for the formation of additional commensurate-order (CO) and incommensurate-order (ICO) chaotic diagrams^43–45^. Also, it investigates multiple influence methods and synchronization schemes constructed to synchronize the connections of various fractional discrete chaotic environments^46,47^. These research investigations revealed that the mechanism’s behaviour is greatly reliant on the fractional-order picked out, highlighting its dynamic and convoluted form, making it an exciting area for research in the discipline of fractional approaches^48,49^. Coccolo and Sanjuán^50^ contemplated the nonlinear delayed forcing drives a non-delayed duffing oscillator. Coccolo et al.^51^ presented the fractional damping effects on the transient dynamics of the duffing oscillator.

In fact, most former satellite model studies have concentrated on classical calculus. Unfortunately, the scientific investigation of DF-satellite models is still insufficient, with little research devoted to investigating their behaviour and attributes. Tsui and Jones^52^ explored the control of higher-dimensional chaos in satellite attitude control problem. Kuang et al.^53^ expounded the chaotic attitude motion of satellites under small perturbation torques. Furthermore, Kuang et al.^54^ contemplated the chaotic dynamics of an asymmetrical gyrostat. Kong et al.^55^ described the control of chaotic attitude motion of a perturbed spacecraft, while the researchers of^56^ investigated controlling and synchronization of a fractional-order chaotic satellite model. The research emphasizes the framework’s challenging and diverse behaviour, emphasizing the importance of fractional aspects in the sophistication and adaptability of satellite models. A great deal of the prior study concentrated mainly on CO theories in continuous-time fractional-order models. Yet it seems that there is a substantial dearth of research regarding the influence of the ICO scenario on the fluidity of these models. Indeed, ICO is a subset of a fractional-order structure defined by revealing the order for which the formula differs. As a result, the simulation’s liberty strengthens. This points to an unresolved issue in the discipline of discrete models, especially within the setting of incommensurate fractional systems. Recognizing the functioning and features of incommensurate fractional satellites may provide significant discoveries and prospective uses in a wide range of fields, including neural structures, technology, artificial intelligence, viscosity, control research, cognitive behaviour and numerous others^57–59^. As a result, additional inquiry and exploration on this subject are required to identify the distinctive features and conceivable advantages of incommensurate fractional satellite models.

Motivated by the prior argumentation, the goal of this article is to investigate and evaluate the dynamic practices of the DF-satellite system, which includes both CO and ICO fractional exponents. By means of an amalgamation of quantitative and qualitative inspections, we execute an extensive review of the key features of this DF-satellite model. We investigate the chaotic behaviour of satellite constellations using multiple techniques, including dissipativity, fixed points, bifurcation illustrations, Poincáare maps and Lyapunov factors. The suggested system’s dissipative nature (strange attractor) is defended. We acquire the proposed model’s fixed points and at every fixed point, we notice that a single of the eigenvalues of the satellite system’s Jacobian matrix is non-negative, confirming the zone of instability. Using the oversight-control procedure, we determine the synchronization of two equivalent satellite constellations. These investigations provide fresh perspectives on the functioning of satellite networks. GPS systems, telecommunications, planet perception and climate prediction can all benefit from measurements. This shows the distinctiveness of our work.

The article is organized as follows: in “Configuration of the DF-satellite model” section, we outline the DF-satellite system and provide key introductory notions concerning DF calculus. “Qualitative analysis of fractional satellite model” section presents a qualitative analysis of the system architecture, focusing on its facts, which is followed by an explanation of the configuration’s design specifications in the second section. “Nonlinear dynamics of the DF-Satellite model” section explores a review of the exciting properties of the DF-satellite model, with emphasis on both CO and ICO cases. The system is dissipative, maximum Lyapunov exponent $(\eta _{\max })$ calculation, bifurcation plots and phase depictions aid in this inquiry. “Control of fractional-order satellite model” section entails applying the sample entropy evaluation (SpEn) to determine variability and verify the existence of chaotic patterns in the system. Furthermore, we suggested adaptable dynamic regulators for the put-forward DF-satellite model’s stability and synchronization. “Conclusion” section ends the work by indicating potential research goals.

## Configuration of the DF-satellite model

The satellite’s attitude dynamics are encoded in the inertial coordinate configuration^60,61^ as

$$\begin{aligned} \dot{\Theta }={\Im }_{\chi _{1}}+{\Im }_{\chi _{2}}+{\Im }_{\chi _{3}}, \end{aligned}$$

where $\Theta$ denotes the aggregate amount of momentum performing on the the spacecraft. The flywheel’s rotational acceleration, gravitational acceleration and disruption torque are denoted by ${\Im }_{\chi _{1}},~ {\Im }_{\chi _{2}}$ and ${\Im }_{\chi _{3}}$, respectively. The entire momentum $\Theta$ defined as

$$\begin{aligned} \Theta =\mathcal {I}\vartheta , \end{aligned}$$

where $\mathcal {I}$ signifies the inertial matrix and $\vartheta$ is the rotational velocity.

The differentiation of the entire momentum $\Theta$ can be described as

$$\begin{aligned} \dot{\Theta }=\mathcal {I}\dot{\vartheta }+\vartheta \times \mathcal {I}\vartheta . \end{aligned}$$

The symbol $\times$ represents the vectors’ cross-product. By equating these formulas, we obtain

$$\begin{aligned} \mathcal {I}\dot{\vartheta }+\vartheta \times \mathcal {I}\vartheta ={\Im }_{\chi _{1}}+{\Im }_{\chi _{2}}+{\Im }_{\chi _{3}}. \end{aligned}$$

Selecting $\mathcal {I}=diag({\mathcal {I}}_{\textbf{u}},{\mathcal {I}}_{\textbf{v}},{\mathcal {I}}_{\textbf{w}})$ such as

$$\begin{aligned} {\Im }_{\chi _{1}}=\begin{pmatrix} {\Im }_{\chi _{1}\textbf{u}}\\ {\Im }_{\chi _{1}\textbf{v}}\\ {\Im }_{\chi _{1}\textbf{w}} \end{pmatrix};~~{\Im }_{\chi _{2}}=\begin{pmatrix} {\Im }_{\chi _{2}\textbf{u}}\\ {\Im }_{\chi _{2}\textbf{v}}\\ {\Im }_{\chi _{2}\textbf{w}} \end{pmatrix};~~ {\Im }_{\chi _{3}}=\begin{pmatrix} {\Im }_{\chi _{3}\textbf{u}}\\ {\Im }_{\chi _{3}\textbf{v}}\\ {\Im }_{\chi _{3}\textbf{w}} \end{pmatrix}. \end{aligned}$$

The satellite model^5^ is referred to as

$$\begin{aligned} {I}_{\textbf{u}}\dot{\vartheta }_{\textbf{u}}\equiv \vartheta _{\textbf{v}}\vartheta _{\textbf{w}}({\mathcal {I}}_{\textbf{v}}-{\mathcal {I}}_{\textbf{w}})+\textbf{g}_{\textbf{u}}+{\textbf{z}}_{\textbf{u}},\\{I}_{\textbf{v}}\dot{\vartheta }_{\textbf{v}}\equiv \vartheta _{\textbf{u}}\vartheta _{\textbf{w}}({\mathcal {I}}_{\textbf{w}}-{\mathcal {I}}_{\textbf{u}})+\textbf{g}_{\textbf{v}}+{\textbf{z}}_{\textbf{v}},\\{I}_{\textbf{w}}\dot{\vartheta }_{\textbf{w}}\equiv \vartheta _{\textbf{u}}\vartheta _{\textbf{v}}({\mathcal {I}}_{\textbf{u}}-{\mathcal {I}}_{\textbf{v}})+\textbf{g}_{\textbf{w}}+{\textbf{z}}_{\textbf{w}}, \end{aligned}$$

where

$$\begin{aligned} \textbf{g}_{\textbf{u}}\equiv \big [({\Im }_{\chi _{1}\textbf{u}}+{\Im }_{\chi _{2}\textbf{u}})+{\Im }_{\chi _{3}\textbf{u}}\big ];~\textbf{g}_{\textbf{v}}\equiv \big [({\Im }_{\chi _{1}\textbf{v}}+{\Im }_{\chi _{2}\textbf{v}})+{\Im }_{\chi _{3}\textbf{v}}\big ];~\textbf{g}_{\textbf{w}}\equiv \big [({\Im }_{\chi _{1}\textbf{w}}+{\Im }_{\chi _{2}\textbf{w}})+{\Im }_{\chi _{3}\textbf{w}}\big ], \end{aligned}$$

Here, $\textbf{g}_{\textbf{u}},~\textbf{g}_{\textbf{v}}$ and $\textbf{g}_{\textbf{w}}$ are internal disturbances torques, while ${\textbf{z}}_{\textbf{u}},~{u_{2}}_{\textbf{v}}$ and ${u_{3}}_{\textbf{w}}$ constitute three influence torques. Consider that ${\mathcal {I}}_{\textbf{w}}<{\mathcal {I}}_{\textbf{v}}<{\mathcal {I}}_{\textbf{u}}.$ Taking ${\mathcal {I}}_{\textbf{u}}=3,~{\mathcal {I}}_{\textbf{v}}=2$ and ${\mathcal {I}}_{\textbf{w}}=1.$ ). These torques are chosen to be sufficiently large to induce chaotic motion and are comparable in magnitude with the available thruster torques. In^52^, the presented values of the “perturbing torques” are arbitrarily selected to make the model chaotic:

$$\begin{aligned} \begin{pmatrix} \textbf{g}_{\textbf{u}}\\ \textbf{g}_{\textbf{v}}\\ \textbf{g}_{\textbf{w}} \end{pmatrix}=\begin{pmatrix} -1.2&0&\sqrt{6}/2\\ 0&0.35&0\\ -\sqrt{6}&0&-0.4 \end{pmatrix}\begin{pmatrix} \vartheta _{\textbf{u}}\\ \vartheta _{\textbf{v}}\\ \vartheta _{\textbf{w}} \end{pmatrix}. \end{aligned}$$

The formula for a three-dimensional in form chaotic satellite model is:

$$\begin{aligned} {\left\{ \begin{array}{ll} \dot{\textbf{u}}=\beta _{\textbf{u}}\textbf{v}\textbf{w} -\frac{1.2}{{\mathcal {I}}_{\textbf{u}}}\textbf{u}+\frac{\sqrt{6}}{2{\mathcal {I}}_{\textbf{u}}}\textbf{w},\\ \dot{\textbf{v}}=\beta _{\textbf{v}}\textbf{u}\textbf{w} +\frac{0.35}{{\mathcal {I}}_{\textbf{v}}}\textbf{v},\\ \dot{\textbf{w}}=\beta _{\textbf{w}}\textbf{u}\textbf{v} -\frac{\sqrt{6}}{{\mathcal {I}}_{\textbf{w}}}\textbf{u}-\frac{\sqrt{0.4}}{2{\mathcal {I}}_{\textbf{w}}}\textbf{w}, \end{array}\right. } \end{aligned}$$

where $\beta _{\textbf{u}}=\frac{{\mathcal {I}}_{\textbf{v}}-{\mathcal {I}}_{\textbf{w}}}{{\mathcal {I}}_{\textbf{u}}},~\beta _{\textbf{v}}=\frac{{\mathcal {I}}_{\textbf{w}}-{\mathcal {I}}_{\textbf{u}}}{{\mathcal {I}}_{\textbf{v}}}$ and $\beta _{\textbf{w}}=\frac{{\mathcal {I}}_{\textbf{u}}-{\mathcal {I}}_{\textbf{v}}}{{\mathcal {I}}_{\textbf{w}}},$ then we have $\beta _{\textbf{u}}=1/3,~\beta _{\textbf{v}}=-1$ and $\beta _{\textbf{w}}=1.$

The satellite model in all three planes has been reformulated as:

2.1 $$\begin{aligned} {\left\{ \begin{array}{ll} \dot{\textbf{u}}\equiv \frac{1}{3}\textbf{v}\textbf{w}-\chi _{1}\textbf{u}+\frac{1}{\sqrt{6}}\textbf{w},\\ \dot{\textbf{v}}\equiv -\textbf{u}\textbf{w}+\chi _{2}\textbf{v},\\ \dot{\textbf{w}}\equiv \textbf{u}\textbf{v}-\sqrt{6}\textbf{u}-\chi _{3}\textbf{w}, \end{array}\right. } \end{aligned}$$

where the values of $\chi _{1}=0.4,~\chi _{2}=0.175$ and $\chi _{3}=0.4.$ Such values were obtained for 50,000 data points on this Poincaré section whenever the motion intersected this hyperplane, as demonstrated in “Nonlinear dynamics of the DF-Satellite model” section. Therefore, this information was then applied to our dynamical modeling and data analysis for the control. Two alternative tactics for dynamic reconstruction of the model were tested: interspike interval simulation and the basic way of applying a specific network parameter to the Poincaré section to recreate the behaviors.

As shown in the formula (2.1), the discrete satellite model displays “memory influence” similar to fractional discrete mechanisms. This indicates that the classical model has the capability of expanding to fractional-order. As a result, using the Caputo formulation results in an innovative fractional discrete satellite model:

2.2 $$\begin{aligned} {\left\{ \begin{array}{ll} \,^{c}\Delta _{\sigma }^{\delta }\textbf{u}(\sigma ) =\frac{1}{3}\textbf{v}(\sigma +\delta -1)\textbf{w} (\sigma +\delta -1)-\chi _{1}\textbf{u}(\sigma +\delta -1)+\frac{1}{\sqrt{6}}\textbf{w}(\sigma +\delta -1),\\ \,^{c}\Delta _{\sigma }^{\delta }\textbf{v}(\sigma ) =-\textbf{u}(\sigma +\delta -1)\textbf{w}(\sigma +\delta -1)+\chi _{2}\textbf{v}(\sigma +\delta -1),\\ \,^{c}\Delta _{\sigma }^{\delta }\textbf{w}(\sigma )= \textbf{u}(\sigma +\delta -1)\textbf{v}(\sigma +\delta -1) -\sqrt{6}\textbf{u}(\sigma +\delta -1)-\chi _{3}\textbf{w}(\sigma +\delta -1), \end{array}\right. } \end{aligned}$$

where $\sigma \in \mathbb {N}_{\textbf{d}-\delta +1}$ and $\delta \in (0,1].$ The Caputo difference formulation $\,^{c}\Delta _{\sigma }^{\delta }\mathcal {W}$ of a mapping $\mathcal {W}(\sigma )$ is described as:

### Definition 2.1

(^28^) The $\delta ^{th}$ fractional sum for a mapping $\mathcal {W}$ can be described as

2.3 $$\begin{aligned} \Delta _{\textbf{d}}^{-\delta }\mathcal {W}(\sigma ) =\frac{1}{\Gamma (\delta )}\sum \limits _{\ell =\textbf{d}}^{\textbf{d} -\delta }(\ell -1-\textbf{d})^{(\delta -1)}\mathcal {W}(\ell ),~~\forall \sigma \in \mathbb {N}_{\textbf{d}+\delta }, \end{aligned}$$

where $\delta >0$ and $\Gamma (.)$ denotes the Gamma function.

### Definition 2.2

(^62^) For $\sigma \in \mathbb {N}_{\textbf{d}+\textbf{n}-\delta },~\delta \notin \mathbb {N}$ and $\textbf{n}=\lceil \delta \rceil +1.$ Suppose there be a Caputo-like difference formula for a mapping $\mathcal {W}(\sigma )$ can be described as:

2.4 $$\begin{aligned} \,^{c}\Delta _{\textbf{d}}^{\delta }\mathcal {W}(\sigma )=\Delta _{\textbf{d}}^{-(\textbf{n}-\delta )}\Delta ^{\textbf{n}}\mathcal {W}(\sigma )=\frac{1}{\Gamma (\textbf{n}-\delta )}\sum \limits _{\ell =\textbf{d}}^{\sigma -(\textbf{n}-\delta )}(\sigma -\ell -1)^{(\textbf{n}-\delta -1)}\Delta ^{\textbf{n}}\mathcal {W}(\ell ), \end{aligned}$$

where $\Delta ^{\textbf{n}}\mathcal {W}(\sigma )$ and $(\sigma -1-\ell )^{(\textbf{n}-\delta +1)}$ represents the $\textbf{n}^{th}$ non-fractional difference formulation and the falling factorial mapping, respectively, presented as:

2.5 $$\begin{aligned} \Delta ^{\textbf{n}}\mathcal {W}(\sigma )=\Delta (\Delta ^{\textbf{n}-1} \mathcal {W}(\sigma ))=\sum \limits _{\kappa =0}^{\textbf{n}} \left( \begin{array}{c}\textbf{n}\\ \kappa \end{array}\right) (-1)^{\textbf{n} -\kappa }\mathcal {W}(\sigma +\kappa ),~~\sigma \in \mathbb {N}_{\textbf{d}}, \end{aligned}$$

and

2.6 $$\begin{aligned} (\sigma -\ell -1)^{(\textbf{n}-\delta -1)}=\frac{\Gamma (\sigma -1)}{\Gamma (\sigma +1-\ell -\textbf{n}+\delta )}. \end{aligned}$$

### Remark 2.1

For $\textbf{n}=1$, we can described the Caputo-type formulation as:

2.7 $$\begin{aligned} \,^{c}\Delta _{\textbf{d}}^{\delta }\mathcal {W}(\sigma )=\Delta _{\textbf{d}}^{-1(1-\delta )} \Delta \mathcal {W}(\sigma )=\frac{1}{\Gamma (1-\delta )}\sum \limits _{\ell =\textbf{d}}^{\sigma -(1-\delta )}(\sigma -1-\ell )^{(-\delta )}\Delta \mathcal {W}(\ell ),~~\sigma \in \mathbb {N}_{\sigma -\delta +1}. \end{aligned}$$

Here, we are able to calculate the mathematical argument for the DF-satellite model (2.2) using the subsequent hypothesis:

### Theorem 2.1

(^43^) The solution of the subsequent fractional difference framework:

2.8 $$\begin{aligned} {\left\{ \begin{array}{ll} \,^{c}\Delta _{\textbf{d}}^{\delta }\mathcal {W}(\sigma )=\textbf{F}\big (\sigma +\delta -1, \mathcal {W}(\sigma +\delta -1)\big ),\\ \Delta ^{\jmath }\mathcal {W}(\sigma )=\mathcal {W}_{\jmath },~~~\textbf{n}=\lceil \delta \rceil +1 \end{array}\right. } \end{aligned}$$

is written as

2.9 $$\begin{aligned} \mathcal {W}(\sigma )=\mathcal {W}_{0}(\sigma )+\frac{1}{\Gamma (\delta )}\sum \limits _{\ell =\textbf{n}-\delta }^{\sigma -\delta }(\sigma +1-\ell )^{(\delta -1)} \textbf{F}\big (\ell -1+\delta ,\mathcal {W}(\ell -1+\delta )\big ),~~\sigma \in \mathbb {N}_{\textbf{d}+\textbf{n}}, \end{aligned}$$

where

2.10 $$\begin{aligned} \mathcal {W}_{0}(\sigma )=\sum \limits _{\jmath =0}^{\textbf{n}-1}\frac{(\sigma -\textbf{d})^{\jmath }}{\Gamma (\jmath +1)}\Delta ^{\jmath }\mathcal {W}(0). \end{aligned}$$

In accordance with the aforesaid result, the numerical illustration of the DF-satellite system (2.2) is as listed below:

2.11 $$\begin{aligned} {\left\{ \begin{array}{ll} {\textbf{u}}_{\zeta }={\textbf{u}}_{0}+\frac{1}{\Gamma (\delta )} \sum \limits _{\ell =0}^{\zeta -1}\frac{\Gamma (\zeta -\ell -1+\delta )}{\Gamma (\zeta -\ell )}\Big (\frac{1}{3}\textbf{v}(\ell )\textbf{w} (\ell )-\chi _{1}\textbf{u}(\ell )+\frac{1}{\sqrt{6}}\textbf{w}(\ell )\Big ),\\ {\textbf{v}}_{\zeta }={\textbf{v}}_{0}+\frac{1}{\Gamma (\delta )} \sum \limits _{\ell =0}^{\zeta -1}\frac{\Gamma (\zeta -\ell -1+\delta )}{\Gamma (\zeta -\ell )}\Big (-\textbf{u}(\ell )\textbf{w}(\ell )+\chi _{2}\textbf{v}(\ell )\Big ),\\ {\textbf{w}}_{\zeta }={\textbf{w}}_{0}+\frac{1}{\Gamma (\delta )} \sum \limits _{\ell =0}^{\zeta -1}\frac{\Gamma (\zeta -\ell -1+\delta )}{\Gamma (\zeta -\ell )}\Big (\textbf{u}(\ell )\textbf{v}(\ell )-\sqrt{6}\textbf{u}(\ell )-\chi _{3}\textbf{w}(\ell )\Big ). \end{array}\right. } \end{aligned}$$

**Figure 1:**
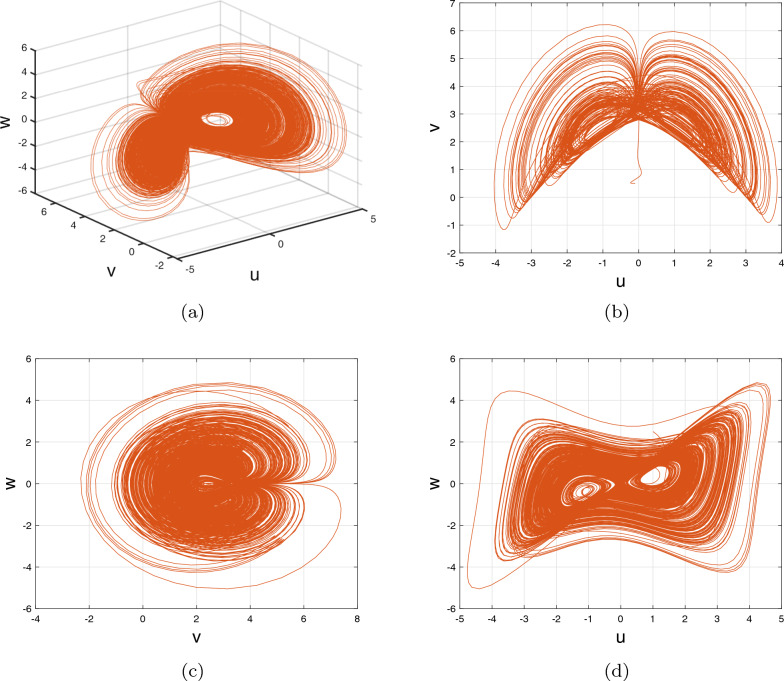
Phase portraits for 3D and 2D for DF-satellite model (2.11) with fractional-order $\delta =0.98$.

Figure 1 depicts the chaotic features for various compartments when fractional order is to be $\delta =0.98$.

Our aim is to boost the extent of the satellite model by implementing the discrete satellite model (2.2) into the system (2.1), resulting in the satellite model shown below.

2.12 $$\begin{aligned} {\left\{ \begin{array}{ll} {\textbf{u}}(\zeta +1)=\frac{1}{3}\textbf{v}(\zeta )\textbf{w}(\zeta )-\chi _{1}\textbf{u} (\zeta )+\frac{1}{\sqrt{6}}\textbf{w}(\zeta ),\\ \textbf{v}(\zeta +1)=-\textbf{u}(\zeta )\textbf{w}(\zeta )+\chi _{2}\textbf{v}(\zeta ),\\ \textbf{w}(\zeta +1)=\textbf{u}(\zeta )\textbf{v}(\zeta )-\sqrt{6}\textbf{u}(\zeta ) -\chi _{3}\textbf{w}(\zeta ). \end{array}\right. } \end{aligned}$$

**Figure 2:**
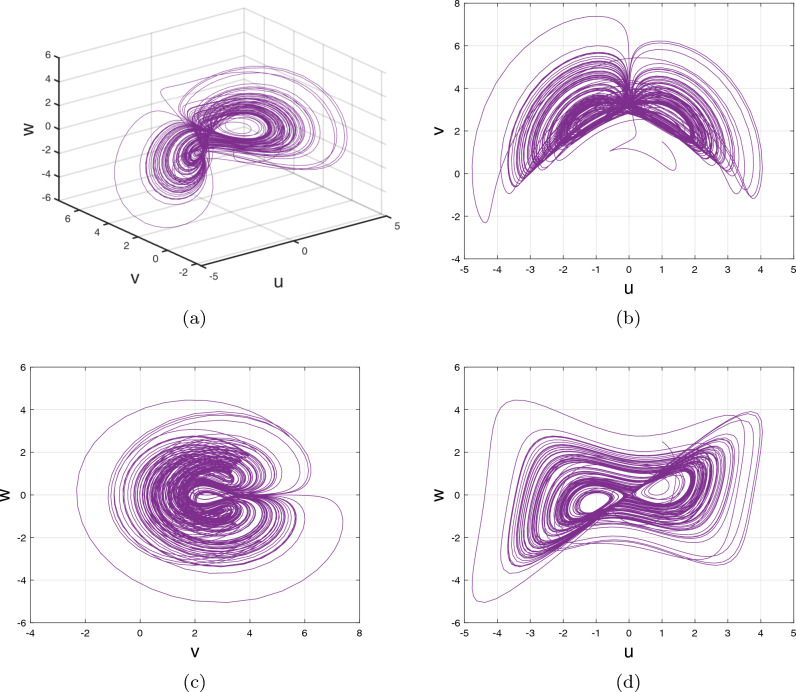
Phase portraits for 3D and 2D for DF-satellite model (2.11) with fractional-order $(\delta _{1},\delta _{2},\delta _{3})=(1,0.53,0.45)$.

Figure 2 indicates that the framework exhibits a chaotic pattern over an important spectrum of fractional factors, particularly throughout the range of $\delta _{\iota }\in (0,1],~~\iota =1,2,3$.

In the current research, we employ the Caputo difference formulation for constructing the fractional-order satellite map from the classical satellite model (2.1). The first-order difference of the satellite system is represented by the following procedure:

2.13 $$\begin{aligned} {\left\{ \begin{array}{ll} \Delta \textbf{u}(\zeta )=\frac{1}{3}\textbf{v}(\zeta )\textbf{w}(\zeta )-\chi _{1}\textbf{u}(\zeta )+\frac{1}{\sqrt{6}}\textbf{w}(\zeta )-\textbf{u}(\zeta ),\\ \Delta \textbf{v}(\zeta )=-\textbf{u}(\zeta )\textbf{w}(\zeta )+\chi _{2}\textbf{v}(\zeta )-\textbf{v}(\zeta ),\\ \Delta \textbf{w}(\zeta )= \textbf{u}(\zeta )\textbf{v}(\zeta )-\sqrt{6}\textbf{u}(\zeta )-\chi _{3}\textbf{w}(\zeta )-\textbf{w}(\zeta ), \end{array}\right. } \end{aligned}$$

where $\Delta \mathcal {W}(\zeta )=\mathcal {W}(\zeta +1)-\mathcal {W}(\zeta )$ indicated the classical difference formulation.

If we replace $\Delta$ in the preceding structure having the Caputo-type formula $\,^{c}\Delta _{\textbf{d}}^{\delta }$ and $\zeta$ into $\varsigma =\sigma +\delta -1$, the resultant arrangement is a fractional-order difference model:

2.14 $$\begin{aligned} {\left\{ \begin{array}{ll} \,^{c}\Delta _{\textbf{d}}^{\delta }\textbf{u}(\sigma )=\frac{1}{3}\textbf{v}(\varsigma )\textbf{w} (\varsigma )-\chi _{1}\textbf{u}(\varsigma )+\frac{1}{\sqrt{6}}\textbf{w}(\varsigma )-\textbf{u}(\varsigma ),\\ \,^{c}\Delta _{\textbf{d}}^{\delta }\textbf{v}(\sigma )=-\textbf{u}(\varsigma )\textbf{w}(\varsigma ) +\chi _{2}\textbf{v}(\varsigma )-\textbf{v}(\varsigma ),\\ \,^{c}\Delta _{\textbf{d}}^{\delta }\textbf{w}(\sigma )= \textbf{u}(\varsigma )\textbf{v}(\varsigma )-\sqrt{6}\textbf{u}(\varsigma )-\chi _{3}\textbf{w}(\varsigma ), \end{array}\right. } \end{aligned}$$

where $\sigma \in \mathbb {N}_{\textbf{d}+1-\delta },$ $\textbf{d}$ is the starting point and $\delta \in (0,1]$ indicates the fractional-order.

## Qualitative analysis of fractional satellite model

This section investigates the requirements for dynamical evaluations of the DF-satellite model (2.14), including dissipatvity of the system, fixed points, inavriancy of the $\textbf{v}$-axis and maximum Lyapunov exponents $\eta _{\max }.$

### Existence of dissipativeness

Here, the vector representation of (2.14) can be described as:

3.1 $$\begin{aligned} \,^{c}\Delta _{\textbf{d}}^{\delta }{X_{1}}(\sigma +1-\delta )=\tilde{\Upsilon }(\sigma +1-\delta )=\begin{pmatrix} \Upsilon _{1}(\textbf{u},\textbf{v},\textbf{w})\\ \Upsilon _{2}(\textbf{u},\textbf{v},\textbf{w})\\ \Upsilon _{3}(\textbf{u},\textbf{v},\textbf{w})\\ \end{pmatrix}, \end{aligned}$$

where $${X_{1}}(\sigma +1-\delta )=(\textbf{u},\textbf{v},\textbf{w})$$ and

3.2 $$\begin{aligned} \tilde{\Upsilon }(\textbf{u})=\begin{pmatrix} \Upsilon _{1}(\textbf{u},\textbf{v},\textbf{w}) =\frac{1}{3}\textbf{v}(\sigma )\textbf{w}(\varsigma )-\chi _{1} \textbf{u}(\sigma )+\frac{1}{\sqrt{6}}\textbf{w}(\sigma )-\textbf{u}(\sigma )\\ \Upsilon _{2}(\textbf{u},\textbf{v},\textbf{w})=-\textbf{u}(\sigma )\textbf{w} (\sigma )+\chi _{2}\textbf{v}(\sigma )-\textbf{v}(\sigma )\\ \Upsilon _{2}(\textbf{u},\textbf{v},\textbf{w})= \textbf{u}(\sigma )\textbf{v} (\sigma )-\sqrt{6}\textbf{u}(\sigma )-\chi _{3}\textbf{w}(\sigma ) \end{pmatrix}, \end{aligned}$$

where $\chi _{1}=0.40,~\chi _{2}=0.175,~\chi _{3}=0.4.$ We examine a particular $\Lambda (\sigma )\in \mathbb {R}^{3}$ domain containing a uniform boundary and $\Lambda (\sigma )=\Theta _{\sigma }(\Lambda )$, where $\Theta _{\sigma }$ is the flow velocity of $\tilde{\Upsilon }$.

Assume that $\mathcal {V}(\sigma )$ indicates the volume of $\Lambda (\sigma ).$

Making the use of Liouville’s theorem, we have

3.3 $$\begin{aligned} \dot{\mathcal {V}}(\sigma )=\int _{\Lambda (\sigma )} (\nabla .\tilde{\Upsilon })d\textbf{u}d\textbf{v}d\textbf{w}. \end{aligned}$$

Thus, the divergence of the satellite model (2.1) is expressed as:

3.4 $$\begin{aligned} \nabla .\tilde{\Upsilon }=\Big [\frac{\partial \Upsilon _{1}}{\partial \textbf{u}}+\frac{\partial \Upsilon _{2}}{\partial \textbf{v}}+\frac{\partial \Upsilon _{3}}{\partial \textbf{w}}\Big ]=-\chi _{1}+\chi _{2}-\chi _{3}=-0.625. \end{aligned}$$

In view of (3.3) and (3.4), we attain the fractional difference equation as:

3.5 $$\begin{aligned} \,^{c}\Delta _{\textbf{d}}^{\delta }\mathcal {V}(\sigma +1-\delta )=-0.625\mathcal {V}(\sigma +1-\delta ), \end{aligned}$$

The solution of (3.5) can be described as:

3.6 $$\begin{aligned} \mathcal {V}(\sigma )=\exp (-0.625 \sigma )\mathcal {V}(0). \end{aligned}$$

Thus, the volumes of the beginning points decreased by $\exp$ in relation to time $\sigma$. $\mathcal {V}(\sigma )\mapsto 0$ when $\sigma \mapsto \infty ,$ $\sigma$ increases at a pace that is exponential. This system’s constraints are confined to the particular limit set that includes zero volume. The strange attractors influence the asynchronous action of a DF-satellite model (3.6). It denotes that the framework (2.14) exhibits chaotic pattern. This supports the existence of dissipative creation in DF-satellite systems (2.14).

### Fixed points

In order to investigate the dynamics of (2.14), we initially obtain the fixed points. For this, first we described the following lemma, which is mainly due to Matignon^63^.

#### Lemma 3.1

(^63^) Assume that there is a fixed point ${X_{1}}_{0}$ of the fractional-order-system and the eigenvalues of Jacobian matrix at the associated fixed points verifies the subsequent assumptions:

$$\begin{aligned} \big \vert \arg \big (eig(\mathcal {J}(\gamma _{\iota }))\big )\big \vert <\delta \pi /2~~~~\implies ~~~~\delta >\max \bigg (\frac{2}{\pi } \arctan \Big (\frac{\Im (\gamma _{\iota })}{\Re (\gamma _{\iota })}\Big )\bigg ). \end{aligned}$$

To identify the fixed points, address the subsequent expressions in (2.14) equating to zero as follows:

3.7 $$\begin{aligned} {\left\{ \begin{array}{ll} 0=\frac{1}{3}\textbf{v}(\varsigma )\textbf{w}(\varsigma )-\textbf{d}\textbf{u}(\varsigma ) +\frac{1}{\sqrt{6}}\textbf{w}(\varsigma )-\textbf{u}(\varsigma ),\\ 0=-\textbf{u}(\varsigma )\textbf{w}(\varsigma )+b_{1}\textbf{v}(\varsigma )-\textbf{v}(\varsigma ),\\ 0= \textbf{u}(\varsigma )\textbf{v}(\varsigma )-\sqrt{6}\textbf{u}(\varsigma )-c_{1}\textbf{w}(\varsigma ), \end{array}\right. } \end{aligned}$$

The expression has the fixed points:

$$\begin{aligned} &  \tilde{\mathbb {E}}_{0}=(0,0,0)^{\textbf{T}},~~\tilde{\mathbb {E}}_{1} =(1.1910,~2.5766,~0.3785)^{\textbf{T}},~~\tilde{\mathbb {E}}_{2}=(0.1582,~-1.3641,~-1.5086)^{\textbf{T}}, \\  &  \tilde{\mathbb {E}}_{3}=(-0.1582,~-1.3641,~1.5086)^{\textbf{T}},~~\tilde{\mathbb {E}}_{4} =(-1.1910,~2.5766,~-0.3785)^{\textbf{T}}. \end{aligned}$$

Therefore, the Jacobian matrix of the system (2.14) is defined as

3.8 $$\begin{aligned} \mathcal {J}(X_{1})=\begin{pmatrix} -\chi _{1}&0.33\textbf{w}&0.33\textbf{v}+1/\sqrt{6}\\ -\textbf{w}&\chi _{2}&-\textbf{u}\\ \textbf{v}-\sqrt{6}&\textbf{u}&-\chi _{3} \end{pmatrix}. \end{aligned}$$

The expression (3.8) at $\tilde{\mathbb {E}}_{0}=(0,0,0)$ can be described as

3.9 $$\begin{aligned} \mathcal {J}_{\tilde{\mathbb {E}}_{0}}=\begin{pmatrix} -0.4&0&0.4082\\ 0&0.175&0\\ -2.45&0&-0.4 \end{pmatrix}. \end{aligned}$$

At $\tilde{\mathbb {E}}_{0}=(0,0,0),$ the eigenvalues $\gamma _{01,02}=-0.40\pm 0.99\iota$ and $\gamma _{03}=0.175,$ demonstrates that $\tilde{\mathbb {E}}_{0}$ is a saddle-focus fixed point. It signifies a unsatble region.

The expression (3.8) at $\tilde{\mathbb {E}}_{1}=(1.1910,~2.5766,~0.3785)$ can be described as

3.10 $$\begin{aligned} \mathcal {J}_{\tilde{\mathbb {E}}_{1}}=\begin{pmatrix} -0.40&0.124&1.26\\ -0.379&0.175&-1.191\\ 0.127&1.191&-0.40 \end{pmatrix}. \end{aligned}$$

At $\tilde{\mathbb {E}}_{1}=(1.1910,~2.5766,~0.3785),$ the eigenvalues $\gamma _{11}=-0.7999,~\gamma _{12}=0.0875+1.2075\iota$ and $\gamma _{13}=0.0875-1.2075\iota ,$ demonstrates that $\tilde{\mathbb {E}}_{1}$ is a saddle-focus fixed point. It signifies an unstable region.

The expression (3.8) at $\tilde{\mathbb {E}}_{2}=(0.1582,~-1.3641,~-1.5086)$ can be described as

3.11 $$\begin{aligned} \mathcal {J}_{\tilde{\mathbb {E}}_{2}}=\begin{pmatrix} -0.40&-0.498&-0.042\\ 1.509&0.175&-0.158\\ -3.814&0.158&-0.40 \end{pmatrix}. \end{aligned}$$

At $\tilde{\mathbb {E}}_{2}=(0.1582,~-1.3641,~-1.5086),$ the eigenvalues $\gamma _{21,22}=0.0875\pm 0.8766\iota$ and $\gamma _{23}=-0.80,$ demonstrates that $\tilde{\mathbb {E}}_{1}$ is a saddle-focus fixed point. It signifies again an unstable region.

**Figure 3:**
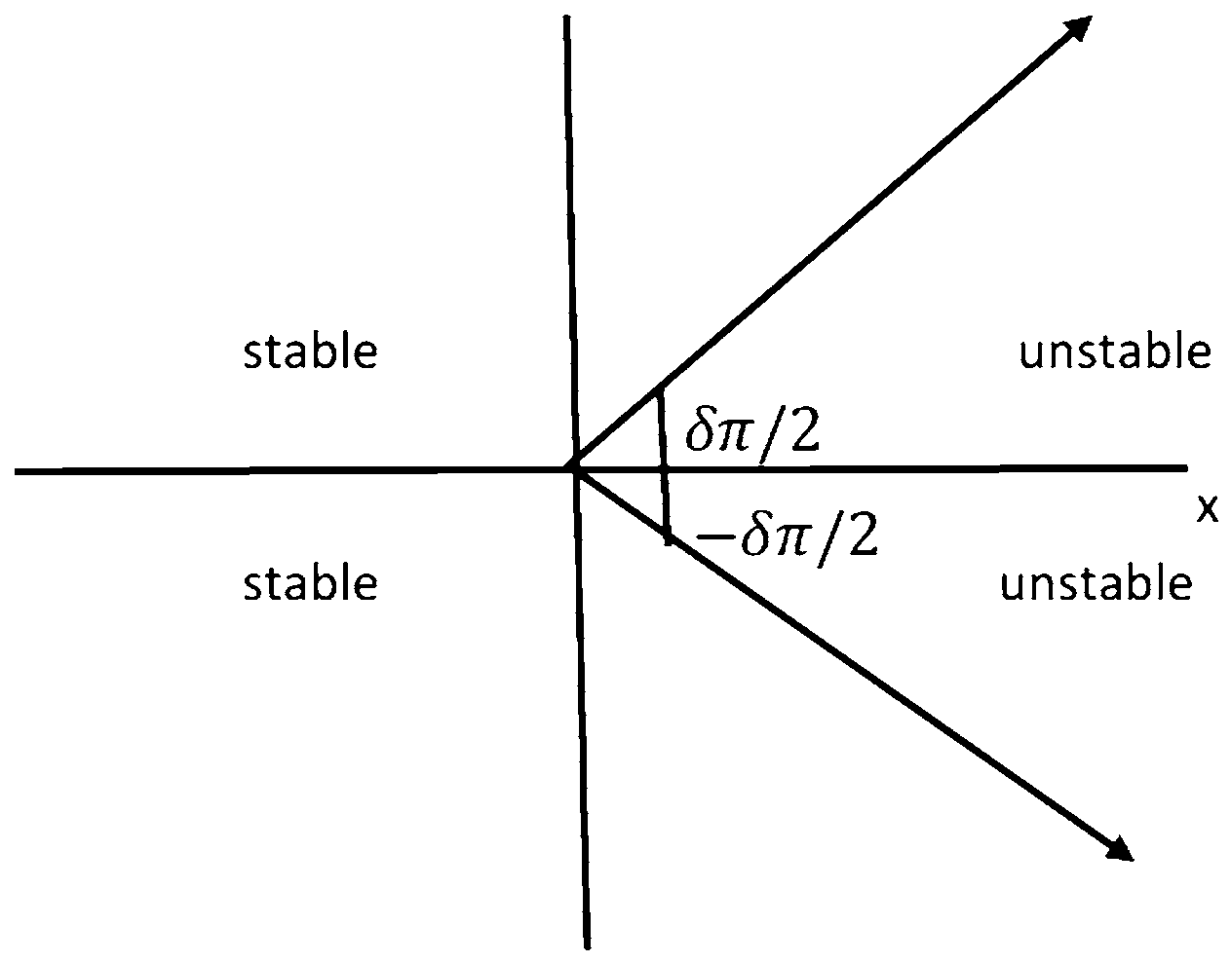
Stabilized zone for fractional-order system.

Analogously, at $\tilde{\mathbb {E}}_{3}=(-0.1582,~-1.3641,~1.5086),$ the eigenvalues $\gamma _{31,32}=0.0875\pm 0.8766\iota$ and $\gamma _{33}=-0.80,$ and $\tilde{\mathbb {E}}_{4}=(-1.1910,~2.5766,~-0.3785),$ the eigenvalues $\gamma _{41}=-0.7999$ and $\gamma _{42,43}=0.0875\pm 1.2075\iota$ demonstrate that $\tilde{\mathbb {E}}_{3}$ and $\tilde{\mathbb {E}}_{4}$ are saddle-focus fixed points. It identifies unstable region. It has been illustrated in Fig. 3.

### The invariancy of $\textbf{v}$-axis

Invariance is crucial for building robust models that can handle variations and uncertainties. By incorporating invariance properties into models or algorithms, we can ensure their performance remains consistent and reliable even under different conditions or inputs. This is particularly important in fields such as computer vision, where recognizing objects or patterns in images requires models to be invariant to changes in scale, rotation, or lighting conditions.

According to DF-satellite model (2.14), it is worth mentioning that when $\textbf{u}(0)=\textbf{w}(0)=0,$ then $\textbf{u}$ and $\textbf{w}$ stay zero $\forall ~\sigma .$ Therefore, $\textbf{v}$-axis signifies an orbit, which can be expressed as

$$\begin{aligned} \,^{c}\Delta _{\sigma }^{\delta }\textbf{v}(\sigma +1-\delta )=\chi _{2}\textbf{v}(\sigma ), \end{aligned}$$

yields

$$\begin{aligned} \textbf{v}(\sigma )=\exp (\chi _{2} \sigma )\textbf{v}(0),~~for~~\textbf{u}=\textbf{w}=0. \end{aligned}$$

As a result, the $\textbf{v}$-axis is an integral component of the unsteady manifold at the starting point of fixed points.

### Maximum Lyapunov exponents ($\eta _{\max }$)

Employing the system parameters for $\chi _{1}=0.4,~\chi _{2}=0.175$ and $\chi _{3}=0.4$, the $\eta _{\max }$ of DF-satellite model (2.14) at $\sigma =100$ can be determined using MATLAB 2023 as: $\mathcal {L}_{1}=0.13959,~\mathcal {L}_{2}=0.00804$ and $\mathcal {L}_{3}=-0.77267.$ When we calculate the $\eta _{\max }$ for the DF-satellite system (2.14), we observe that one is non-negative, other is negative, and one is generally zero, indicating an essential prerequisite for system chaos. It proves that the satellite models are chaotic. Figure 2 depicts it. Here, $\mathcal {L}_{1}=0.13959$ is the $\eta _{\max }$ of satellite system (2.14). The total number of LEs is calculated as $\mathcal {L}_{1}+\mathcal {L}_{2}+\mathcal {L}_{3}=-0.604<0.$ Finally, satellite model (2.14) is dissipative.

## Nonlinear dynamics of the DF-Satellite model

In the following part, the focus is on the novel investigation of how the DF-Satellite model (2.14) behaves. The evaluation will include both CO and ICOs. We will use a variety of computational resources for displaying phase portraits, illustrating bifurcations and calculating the maximum Lyapunov exponent $\eta _{\max }$.

### Commensurate DF-Satellite model

In this section, we will elaborate on the various properties of the CO for the DF-satellite system (2.14). It is essential to comprehend that a CO fractional system consists of formulas with similar orders. To achieve this, we shall subsequently provide the numerical calculation, which originates from Theorem 2.1 and will be provided as follows:

4.1 $$\begin{aligned} {\left\{ \begin{array}{ll} \textbf{u}(\ell )=\textbf{u}(0)+\sum \limits _{\kappa =1}^{\ell } \frac{\Gamma (\ell -\kappa -1+\delta )}{\Gamma (\delta )\Gamma (\ell -\kappa )}\Big [\frac{1}{3}\textbf{v}(\kappa )\textbf{w}(\kappa ) -\chi _{1}\textbf{u}(\kappa )+\frac{1}{\sqrt{6}}\textbf{w}(\kappa )\Big ],\\ \textbf{v}(\ell )=\textbf{v}(0)+\sum \limits _{\kappa =1}^{\ell } \frac{\Gamma (\ell -\kappa -1+\delta )}{\Gamma (\delta )\Gamma (\ell -\kappa )}\Big [-\textbf{u}(\kappa )\textbf{w}(\kappa )+\chi _{2}\textbf{v}(\kappa )\Big ],\\ \textbf{w}(\ell )=\textbf{w}(0)+\sum \limits _{\kappa =1}^{\ell } \frac{\Gamma (\ell -\kappa -1+\delta )}{\Gamma (\delta )\Gamma (\ell -\kappa )}\Big [\textbf{u}(\kappa )\textbf{v}(\kappa )-\sqrt{6}\textbf{u}(\kappa )-\chi _{3}\textbf{w}(\kappa )\Big ]. \end{array}\right. } \end{aligned}$$

**Figure 4:**
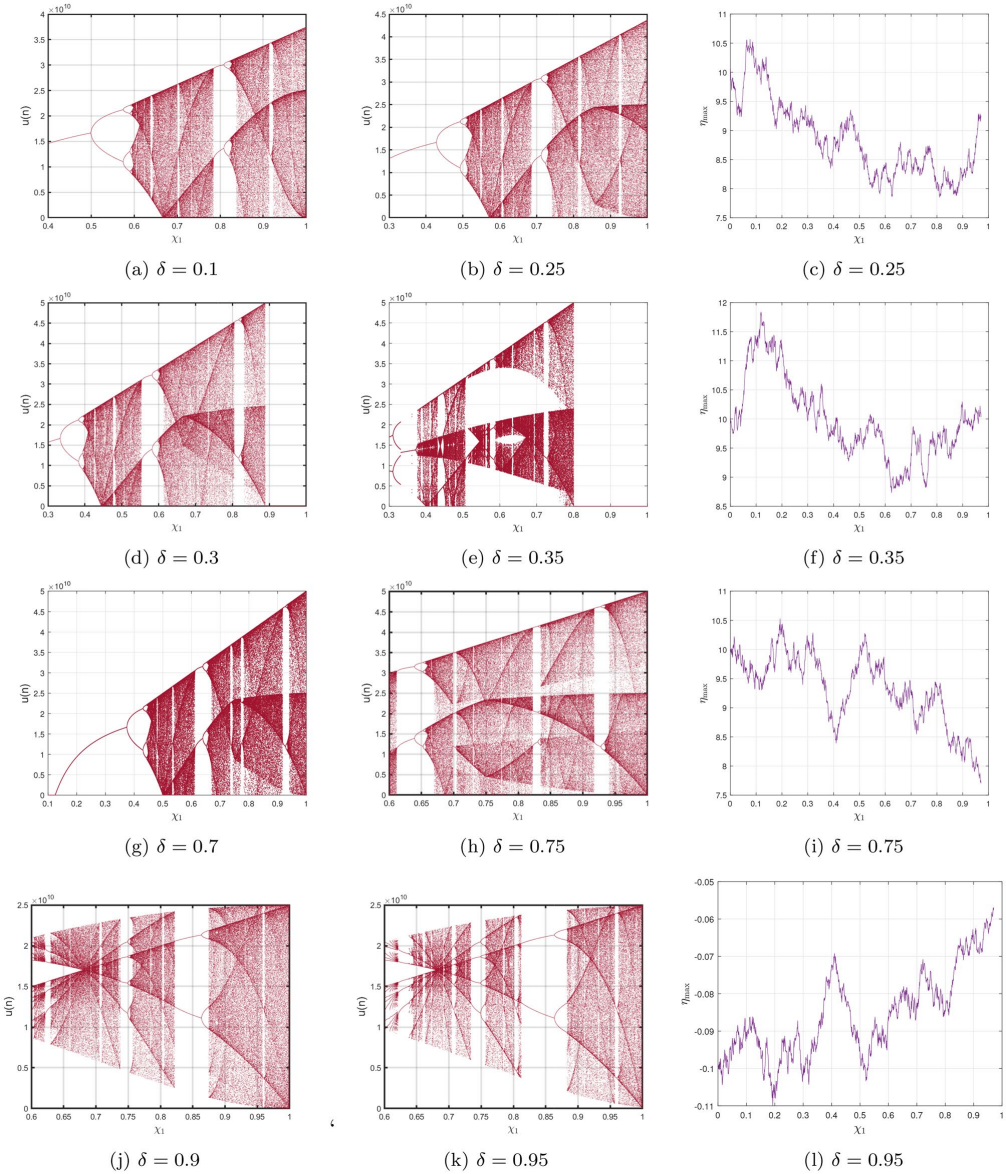
Bifurcation and $\eta _{\max }$ depictions for the CO DF-satellite model. (4.1) when $\chi _{1}\in (0,1)$.

Choosing $\textbf{u}(0)=2.5,~\textbf{v}(0)=1.5,~\textbf{w}(0)=-1.5$ and by varying $\chi _{1}$ from 0 to 1 with the step size $\Delta \chi _{1}=0.001$, we visualize three bifurcations of (4.1), which connect to the C-Os $\delta =0.1,~\delta =0.25,~\delta =0.0.25$ as illustrated in Fig. 4a–c. The parameter’s structures and the CO $\delta$ clearly influence the configurations of the CO DF-satellite model (4.1). In fact, as the CO $\delta$ and parameters of the system decline, the CO DF-satellite model (4.1) exhibits an increasingly large chaotic domain. As a result, increasingly complicated resonances develop, and the mechanism’s behaviour grows more unpredictable. The interaction between DF order and framework variables has an enormous effect on dynamic behaviour, and such modifications may result in a broader spectrum of chaotic structure and convoluted pathways that comprise the DF-satellite model (4.1).

**Figure 5:**
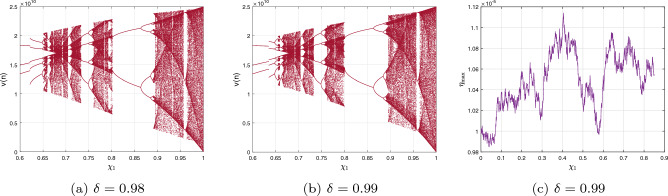
Bifurcation and $\eta _{\max }$ depictions for the C-O DF-satellite model (4.1) when $\delta =0.98$ and $\delta =0.99,$ respectively.

**Figure 6:**
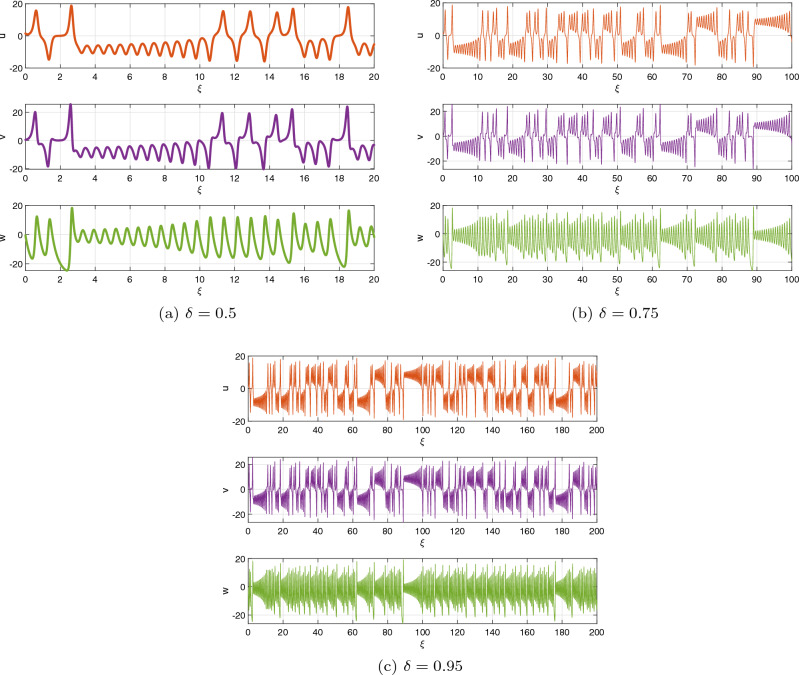
Time-dependent plots for CO DF-satellite model (4.1) for $\delta =0.5,~0.75$ and $\delta =0.95$.

**Figure 7:**
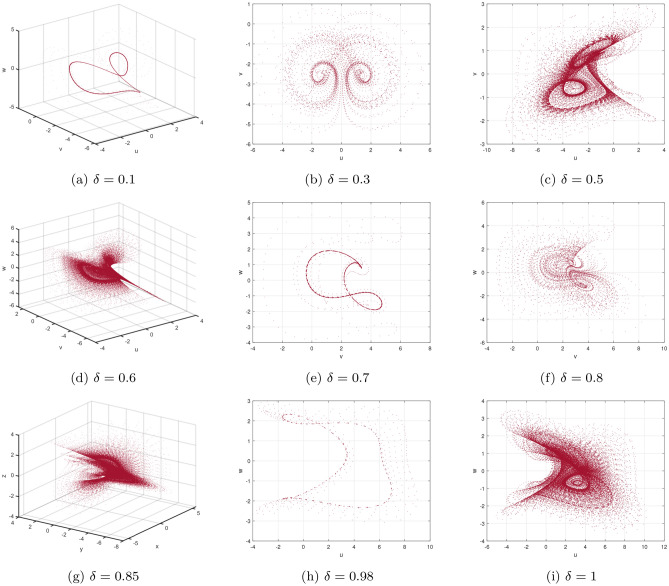
Phase depictions of (4.1) for various fractional-order $\delta$ (**a**) $\delta =0.1$ (**b**) $\delta =0.3,$ (**c**) $\delta =0.5,$ (**d**) $\delta =0.6,$ (**e**) $\delta =0.7,$ (**f**) $\delta =0.8,$ (**g**) $\delta =0.85,$ (**h**) $\delta =0.98,$ (**i**) $\delta =1$.

Presently, alongside $\delta$ as the significant parameter, the bifurcation illustration can be utilized to show the changes in the behaviours of the commensurate DF-satellite model (4.1) as the $\chi _{1}$ fluctuates from 0 to 1 via an increment of 0.001. The bifurcation and the $\eta _{\max }$ are depicted in Fig. 4d–f. We are able to observe that modifying the CO investigates an extensive variety of unpredictable features (chaotic and periodic) of the fractional model in relation to the CO $\delta$. In particular, there are two types of domains in which the system is chaotic and domains in which the motion is oscillatory frequently. When $\delta \in (0.6,0.35),$ recurring views alongside various period orbits show up accompanied by an insignificant chaotic movement in the time range (0.35, 0.75) (see Fig. 4g–i). We can see variations within chaotic and consistent pathways in the configurations of the CO DF-satellite when $\delta \in (0.35,0.75).$ The $\eta _{\max }$ varies within negative and non-negative readings throughout this interval, demonstrating adjustments within chaos and non-chaotic behaviours in the framework. The pathways of the CO DF-satellite model (4.1) indicate chaotic behaviour when the CO $\delta$ is between (0.35, 0.75). Nevertheless, as $\delta$ approaches (0.70, 0.99),  various periods show orbit revolution, demonstrating the framework’s equilibrium  (see Fig. 4i,j). Following that, for greater amounts of $\delta$, Fig. 5a–c presents bifurcation gestures comeback, with an upsurge in the $\eta _{\max }$, demonstrating irregularities throughout the pathways of the CO DF-satellite model (4.1) for $\delta =0.98$ and 0.99,  respectively. The $\eta _{\max }$ in Fig. 6a–c confirms the outlined behavioural characteristics, delivering supplementary proof for the framework’s multifaceted and intricate behaviour and affirming the system’s responsiveness to adjustments in the CO value $\delta =0.98$ and $\delta =0.99,$ respectively. Moreover, considering the $\eta _{\max }$, it is possible to determine that in situations where the $\eta _{\max }$ is negative, the corresponding DF-satellite model (4.1) displays periodic fluctuations. Whenever the order is non-negative, the existence of chaotic fluctuations is deduced. Figure 6a–c depicts the isolated time progression of the configurations $\textbf{u},~\textbf{v}$ and $\textbf{w}$ in the proposed commensurate map to provide an extensive overview of these features for $\delta =0.5,~0.75,~0.99,$ respectively. Figure 7a–i also shows phase representations for various quantities of the CO $(\delta =0.1,~0.3,~0.5,~0.6,~0.7,~0.8,~0.85,~0.98,~1).$ The pathways noticed within the identified commensurate system transform into chaotic fluctuations and recurring behaviours as the CO $\delta$ fluctuates, as shown in the diagrams. The findings highlight the mechanism’s responsiveness to alterations in $\delta$ and indicate the extensive and intricate nature of the constantly changing features in the DF-satellite CO model (4.1).

### Incommensurate DF-Satellite model

The interactions of the ICO DF-satellite model are investigated in this part of the article. ICO requires employing distinctive fractional-orders to feed every formula in the structure. The ICO DF-satellite model is represented in the following manner:

4.2 $$\begin{aligned} {\left\{ \begin{array}{ll} \,^{c}\nabla _{\sigma }^{\delta _{1}}\textbf{u}(\sigma ) =\frac{1}{3}\textbf{v}(\xi )\textbf{w}(\xi )-\chi _{1}\textbf{u}(\xi )+\frac{1}{\sqrt{6}}\textbf{w}(\xi ),\\ \,^{c}\nabla _{\sigma }^{\delta _{2}}\textbf{v}(\sigma ) =-\textbf{u}(\xi )\textbf{w}(\xi )+\chi _{2}\textbf{v}(\xi ),\\ \,^{c}\nabla _{\sigma }^{\delta _{3}}\textbf{w}(\sigma ) = \textbf{u}(\xi )\textbf{v}(\xi )-\sqrt{6}\textbf{u}(\xi )-\chi _{3}\textbf{w}(\xi ), \end{array}\right. } \end{aligned}$$

In view of Theorem 2.1, we are able to convey a mathematical representation of the ICO DF-satellite model (4.2) in the following manner:

4.3 $$\begin{aligned} {\left\{ \begin{array}{ll} \textbf{u}(\ell )=\textbf{u}(0)+\sum \limits _{\kappa =1}^{\ell } \frac{\Gamma (\ell -\kappa -1+\delta _{1})}{\Gamma (\delta _{1}) \Gamma (\ell -\kappa )}\Big [\frac{1}{3}\textbf{v}(\kappa )\textbf{w} (\kappa )-\chi _{1}\textbf{u}(\kappa )+\frac{1}{\sqrt{6}}\textbf{w}(\kappa )\Big ],\\ \textbf{v}(\ell )=\textbf{v}(0)+\sum \limits _{\kappa =1}^{\ell } \frac{\Gamma (\ell -\kappa -1+\delta _{2})}{\Gamma (\delta _{2}) \Gamma (\ell -\kappa )}\Big [-\textbf{u}(\kappa )\textbf{w}(\kappa )+\chi _{2}\textbf{v}(\kappa )\Big ],\\ \textbf{w}(\ell )=\textbf{w}(0)+\sum \limits _{\kappa =1}^{\ell }\frac{\Gamma (\ell -\kappa -1+\delta _{3})}{\Gamma (\delta _{3})\Gamma (\ell -\kappa )}\Big [\textbf{u}(\kappa ) \textbf{v}(\kappa )-\sqrt{6}\textbf{u}(\kappa )-\chi _{3}\textbf{w}(\kappa )\Big ]. \end{array}\right. } \end{aligned}$$

**Figure 8:**
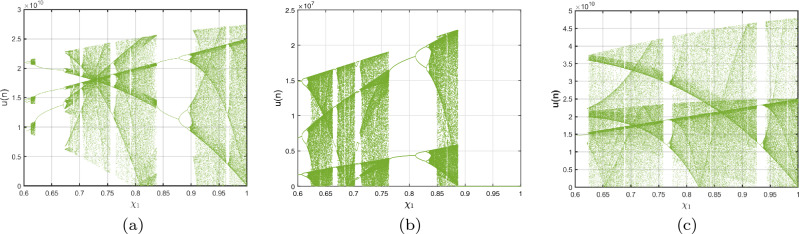
Bifurcations illustration of (4.3) in view of the ICO values (**a**) $(\delta _{1},\delta _{2},\delta _{3})=(1,0.2,0.2),$ (**b**) $(\delta _{1},\delta _{2},\delta _{3})=(0.2,0.6,0.2)$, (**c**) $(\delta _{1},\delta _{2},\delta _{3})=(0.2,0.2,0.85)$.

We investigate the processes and distinctive features of this visualization for the purpose of comprehending their peculiar behaviour and investigating the consequences of using distinguished fractional-orders in the system’s dynamics equations. Figure 8a–c shows three bifurcation diagrams that show the behaviour of the ICO DF-satellite model (4.3) as the value of $\chi _{1}$ fluctuates between (0, 1]. The modelling exercises were performed with the system settings and the ICs $\big (\textbf{u}(0),\textbf{v}(0),\textbf{w}(0)\big )$ set to $(1.5,0.5,-0.5).$ These schematics clearly show distinguished trends, pointing out that modifications in fractional-orders $(\delta _{1},\delta _{2},\delta _{3})$ have an enormous effect on the configurations of the ICO DF-satellite model (4.3). For example, whenever $(\delta _{1},\delta _{2},\delta _{3})=(1,0.2,0.2),$ the structure’s contends develop via repeated to chaotic, using recurring expanding bifurcation when $\chi _{1}$ improves. However, when $(\delta _{1},\delta _{2},\delta _{3})=(0.2, 0.6, 0.2)$, an oscillatory trajectory is noticed, using pathways that stay reliable to earn minimal measurements of $\chi _{1}$ and transforming into chaos as $\delta _{1}$ gets closer to 1. In the scenario of $(\delta _{1},\delta _{2},\delta _{3})=(0.2, 0.2, 0.85),$ a chaotic region is visible all along the range, with the exception of a few confined areas where the framework demonstrates frequent fluctuations, particularly as $\delta _{1}$ decreases towards 0. Additional research was additionally performed in three particular situations to offer a more comprehensive example of the impacts of ICOs on the behaviour of the DF-satellite model (4.3). Such inquiries provide an improved comprehension of how fractional-orders affect the functioning of systems and emphasize the significance of taking ICOs into account when analyzing simulation behaviour.

**Figure 9:**
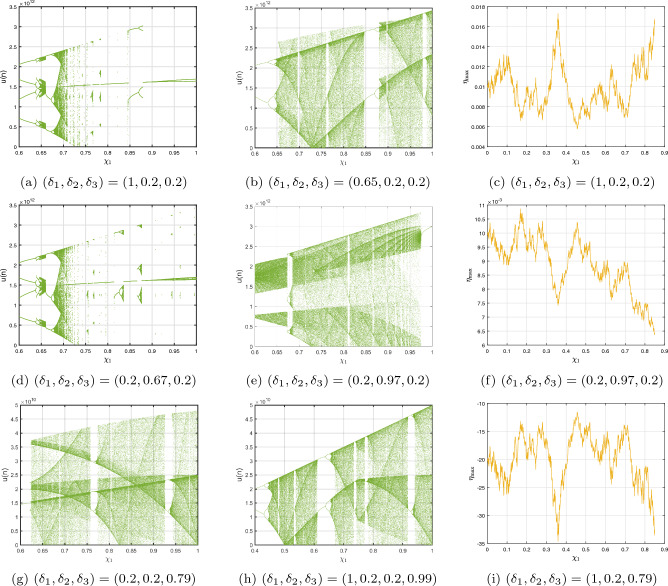
Bifurcation and $\eta _{\max }$ of (4.3) for various ICO.

*Case I*: Figure 9a–c show the change with respect to $\delta _{1}$ via 0 to 1 using an incremental dimension of $\Delta \delta _{1}=0.005$. The bifurcation and associated $\eta _{\max }$ of the ICO DF-satellite model (4.3) for $\delta _{2}=\delta _{3}=0.2$ and the system settings with the ICs $\big (\textbf{u}(0),\textbf{v}(0),\textbf{w}(0)\big )$ set to $(1.5,0.5,-0.5)$ are shown in these illustrations. Figure 9b shows that the configuration of the ICO DF-satellite model (4.3) demonstrates chaotic behaviour for less extensive variations in $\delta _{1}$, as indicated by non-negative $\eta _{\max }$. When $\delta _{1}$ falls within (0.6, 1), the $\eta _{\max }$ shown in Fig. 9c swings within non-negative and negative regions. Through the appearance of regular apertures, this result suggests the existence of chaotic behaviour. As the ICO $\delta _{1}$ expands, paths are transformed from chaotic to consistent movement, which is characterized by orbits that revolve, in which the configurations of the ICO DF-satellite model (4.3) get steady.

*Case II:* The bifurcation illustration and its $\eta _{\max }$ are displayed for studying the fluctuating behaviours of the ICO DF-satellite model (4.3) via $\delta _{2}$ becoming a configurable factor, as shown in Fig. 9d–f. The modelling steps are carried out by differing $\delta _{2}$ in the interval (0, 1) whereas maintaining the ICOs $\delta _{1}=\delta _{3}=0.2$, ICs $\big (\textbf{u}(0),\textbf{v}(0),\textbf{w}(0)\big )=(1.5,0.5,-0.5)$ and system settings remains consistent. The illustration shows that while the order $\delta _{2}$ improves to higher figures, the patterns of motion get less unstable. As $\delta _{2}$ declines, chaotic practices that have elevated $\eta _{\max }$ values show up in Fig. 9e,f, in addition to the emergence of relatively small regular zones via adverse $\eta _{\max }$ parameters. Furthermore, as $\delta _{2}$ falls more thoroughly closer to zero, the $\eta _{\max }$ values fall, after which they come to zero. This is consistent with the appearance of recurring pathways and the evolution of the incommensurate DF-satellite model (4.3) regarding chaos to stable decisions. The identified modifications in the $\eta _{\max }$ and the accompanying shifting trends demonstrate the mechanism’s response to fractional-order $\delta _{2}$ alternatives, pointing out the intricate nature and adaptability of the ICO DF-satellite model (4.3).

**Figure 10:**
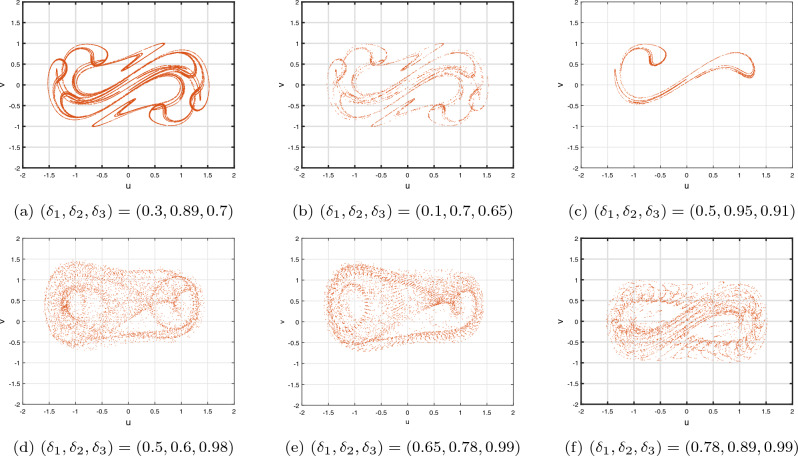
Phase illustrations of (4.3) for various ICO.

*Case III:* Figure 9g–i depicts the bifurcated diagram and the accompanying $\eta _{\max }$ of the identified novel ICO DF-satellite model (4.3) using the parameter $\delta _{3}$ fluctuated between 0 and 1. We preserve the ICOs as $\delta _{1}=\delta _{2}=0.2$ in the present calculation. Figure 9g shows that, in contrast to the earlier instances, the pathways of the incommensurate system reveal chaotic behaviour as the position of $\delta _{3}$ increases, which is illustrated by greater $\eta _{\max }$ parameters. We also observe that as $\delta _{3}$ arrives at 1, the map indicates evolution stipulates and the paths deviate towards infinity, as shown in Fig. 9h. As an instance, if $\delta _{3}=0.923$ and following a certain quantity of repetitions, particularly $\chi _{3}$, the pathways deviate towards infinity. As $\delta _{3}$ decreases, the $\eta _{\max }$ decreases likewise (see Fig. 9i), ultimately achieving the lowest possible significance, resulting in less chaotic and, as a result, more predictable interactions of the model’s indicates. These findings highlight the incommensurate DF-satellite (4.3) responsiveness to fluctuations in order $\delta _{3}$, which leads to an extensive spectrum of flexible actions involving chaotic and cyclical movements. This emphasizes the importance of ICOs in determining the behaviour of the framework. Furthermore, as can be seen in Fig. 10, the phase depictions of the configuration factors of the incommensurate DF-satellite model (4.3), promote the idea that ICOs more precisely symbolize the structure’s behavioural patterns. To sum up, the research highlights the complex and varied characteristics of the ICO DF-satellite model (4.3) and it also highlights the importance of fractional-order selection in modelling and characterizing its fluctuations.

### The sample entropy evaluation (SpEn)

Without a prior understanding of the mechanism that generated the dataset, the aim of sample entropy (SpEn) and approximation entropy (ApEn) is to determine the unpredictability of a sequence of data. For the basic notions and details of the algorithms that have been a vast range of applications and employed in numerous research domains (see^64^).

In the present research, we are employing the SpEn technique to determine the intricate nature of the commensurate and ICO DF-satellite models (2.14) and (4.2), respectively. In contrast to approximate entropy (ApEn), SpEn is capable of accurately measuring the unpredictability of data sets, irrespective of the integrating measurements $(\jmath )$ or their resemblance factor $(\xi )$. As a result, SpEn provides an additional trustworthy and neutral quantifier than SpEn^65^. The SpEn information demonstrates the period of the series’ uncertainty threshold via greater amounts associated with greater variability^66^. The following method of SpEn is calculated:

We begin by defining $\zeta -\jmath +1$ vectors in the following way:

4.4 $$\begin{aligned} \mathcal {W}(\iota )=\big [\vartheta _{1}(\iota ),\ldots ,\vartheta _{1}(\iota +\textbf{n}-1)\big ], \end{aligned}$$

$\forall ~\iota \in [1,\zeta -\jmath +1],$ where $\mathcal {W}(\iota )$ denotes the collection of discrete data points $\vartheta _{1}(1),\vartheta _{1}(2),\ldots \vartheta _{1}(n_{1}).$ Furthermore, we define the subsequent formula as:

4.5 $$\begin{aligned} \mathcal {B}_{\iota }^{\jmath }(\xi )=\frac{\mathcal {K}}{\zeta -\jmath +1}, \end{aligned}$$

where $\mathcal {K}$ denotes a value of $\mathcal {W}(\iota )$ with $d_{1}\big (\mathcal {W}(\iota ),\mathcal {W}(\ell )\big )\le \xi$. At this point, we take $\jmath =2$ and $\xi =0.2std(\mathcal {W})$, where $std(\mathcal {W})$ is the data’s standard deviation. In conceptual terms, the SpEn can be determined as follows:

4.6 $$\begin{aligned} SpEn=-\log \frac{\Upsilon ^{\jmath +1}(\zeta )}{\Upsilon ^{\jmath }(\zeta )}, \end{aligned}$$

where $\Upsilon ^{\jmath }(\xi )$ can be written as

4.7 $$\begin{aligned} \Upsilon ^{\jmath }(\xi )=\frac{1}{\zeta -\jmath +1}\sum \limits _{\iota =1}^{\zeta -\jmath +1}\log \mathcal {B}_{\iota }^{\jmath }(\xi ). \end{aligned}$$

**Figure 11:**
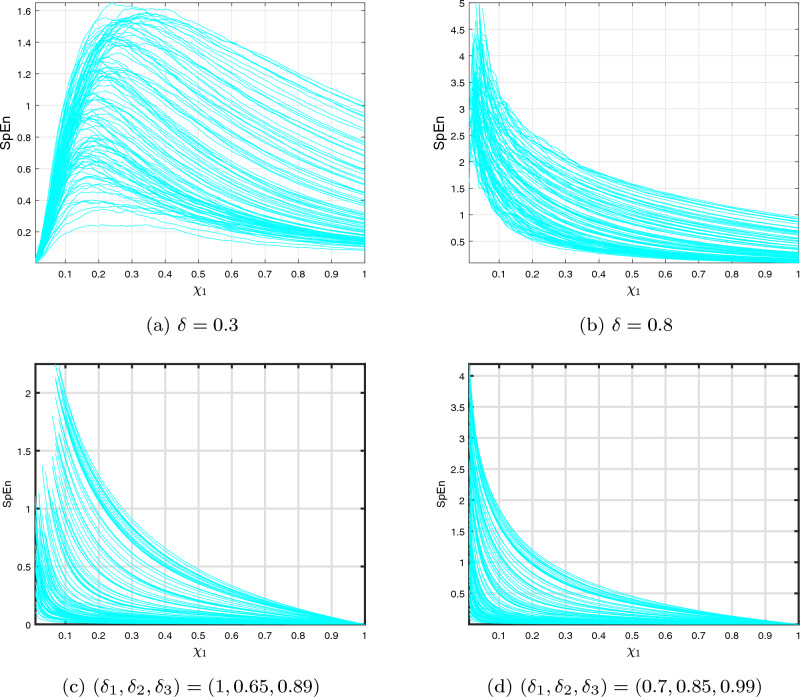
Plots on SpEn of (2.14) and (4.2) for various ICOs.

Here, Fig. 11a–d depicts the SpEn outcomes for the commensurate and the ICO DF-satellite model (2.14) and (4.3) having ICs of $\big (\textbf{u}(0),\textbf{v}(0),\textbf{w}(0)\big )=(1.5,0.5,-0.5).$ The calculated SpEn data represent the time evolution of the system complexity phases, with more significant values indicating greater intricacy. The outcomes show that each of the commensurate and incommensurate DF-satellite model (2.14) and (4.3), respectively, possesses greater intricacy, as evidenced by more substantial SpEn parameters. The findings presented are consistent with the $\eta _{\max }$ examination, verifying the unpredictable character of the processes in the suggested non-integer mechanism. SpEn has several advantages over ApEn. In this case, it is less sensitive to the length of the time series and has better statistical properties. It provides a more stable measure of complexity, even with shorter data sequences. The additional variability and chaotic interactions of the suggested DF-satellite model reinforce the importance of fractional-orders in documenting their extensive interactions. Ultimately, we are able to determine that the SpEn assessment is a successful instrument for precisely determining the level of abstraction of the suggested model.

## Control of fractional-order satellite model

In this section, we will present the stabilization and synchronized state of the satellite model (2.2).

### Stabilization of fractional-order satellite model

A stabilization control system has been proposed in this research to stabilize the suggested DF-satellite chaotic model. The primary objectives of the stabilization approach are to create a powerful responsive device that inspires every instance of the representation asynchronously closer to zero.

To accomplish this, we will review the stability criteria for the fractional-order system.

#### Theorem 5.1

(^67^) Suppose that $\vartheta (\zeta )=\big (\vartheta _{1}(\zeta ),\ldots ,\vartheta _{n_{1}}(\zeta )\big )^{\textbf{T}}$ and $\Phi \in \mathbb {M}_{n_{1}}(\mathbb {R}).$ The linear fractional-order discrete system’s having zero steady state:

5.1 $$\begin{aligned} \,^{c}\Delta _{\textbf{d}}^{\delta }\vartheta (\zeta )=\Phi \vartheta (\varsigma ), \end{aligned}$$

$\forall ~\zeta \in \mathbb {N}_{\textbf{d}-1+\delta }$ is asymptotically stable if:

5.2 $$\begin{aligned} \gamma _{\iota }\in \bigg \{\gamma \in \mathcal {B}:\vert \gamma \vert <\Big (2\cos \frac{\vert arg\gamma \vert -\pi }{2-\delta }\Big )^{\delta }~~and~~\vert arg\gamma \vert >\frac{\delta \pi }{2}\bigg \}, \end{aligned}$$

where $\gamma _{\iota }$ signified the eigenvalue.

Currently, we consider the regulated DF-satellite model which can be expressed as:

5.3 $$\begin{aligned} {\left\{ \begin{array}{ll} \,^{c}\Delta _{\textbf{d}}^{\delta }\textbf{u}(\sigma )=\frac{1}{3} \textbf{v}(\varsigma )\textbf{w}(\varsigma )-\chi _{1}\textbf{u}(\varsigma ) +\frac{1}{\sqrt{6}}\textbf{w}(\varsigma )-\textbf{u}(\varsigma )+\mathcal {B}_{1}(\varsigma ),\\ \,^{c}\Delta _{\textbf{d}}^{\delta }\textbf{v}(\sigma )=-\textbf{u}(\varsigma )\textbf{w}(\varsigma ) +\chi _{2}\textbf{v}(\varsigma )-\textbf{v}(\varsigma )+\mathcal {B}_{2}(\varsigma ),\\ \,^{c}\Delta _{\textbf{d}}^{\delta }\textbf{w}(\sigma )= \textbf{u}(\varsigma )\textbf{v}(\varsigma ) -\sqrt{6}\textbf{u}(\varsigma )-\chi _{3}\textbf{w}(\varsigma )+\mathcal {B}_{3}(\varsigma ),\end{array}\right. } \end{aligned}$$

where $\varsigma =\sigma +\delta -1$ and $\mathcal {B}=(\mathcal {B}_{1},\mathcal {B}_{2},\mathcal {B}_{3})^{\textbf{T}}$ is the adaptive regulate system. The regulation principles introduced in the subsequent proof are geared at stabilizing the suggested innovative DF-satellite model.

#### Theorem 5.2

If appropriate control principles are implemented as outlined below:

5.4 $$\begin{aligned} {\left\{ \begin{array}{ll} \mathcal {B}_{1}(\varsigma )=-\frac{1}{3}\textbf{v}(\varsigma ) \textbf{w}(\varsigma )+\chi _{1}\textbf{u}(\varsigma )-\frac{1}{\sqrt{6}}\textbf{w}(\varsigma ),\\ \mathcal {B}_{1}(\varsigma )=\textbf{u}(\varsigma )\textbf{w}(\varsigma )-\chi _{2}\textbf{v}(\varsigma ),\\ \mathcal {B}_{3}(\varsigma )= -\textbf{u}(\varsigma )\textbf{v}(\varsigma )+\sqrt{6}\textbf{u}(\varsigma ) +\chi _{3}\textbf{w}(\varsigma ).\end{array}\right. } \end{aligned}$$

Subsequently, at its steady state, the fractional-order satellite model can be stabilized.

#### Proof

By replacing $\mathcal {B}_{1},~\mathcal {B}_{2}$ and $\mathcal {B}_{3}$ into (5.3) results in the linear structure shown below:

5.5 $$\begin{aligned} \,^{c}\Delta _{\textbf{d}}^{\delta }\mathcal {W}(\zeta )=\Phi \mathcal {W}(\varsigma ), \end{aligned}$$

where $$\mathcal {W}=(\textbf{u},\textbf{v},\textbf{w})^{\textbf{T}}$$ and

5.6 $$\begin{aligned} \Phi =\begin{pmatrix}-1&0&0\\ 0&-1&0\\ 0&0&0-1 \end{pmatrix} \end{aligned}$$

Clearly, the eigenvalue of (5.5) valid for $\gamma _{\jmath }=1<\Big (2\cos \frac{\vert arg\gamma _{\jmath }\vert -\pi }{2-\delta }\Big )^{\delta }~~and~~\vert arg\gamma _{\jmath }\vert =\pi >\frac{\delta \pi }{2},~~\jmath =1,2,3.$As a result, Theorem 5.1 shows that the regulated fractional-order-satellite model is asymptotically stable. $\square$

**Figure 12:**
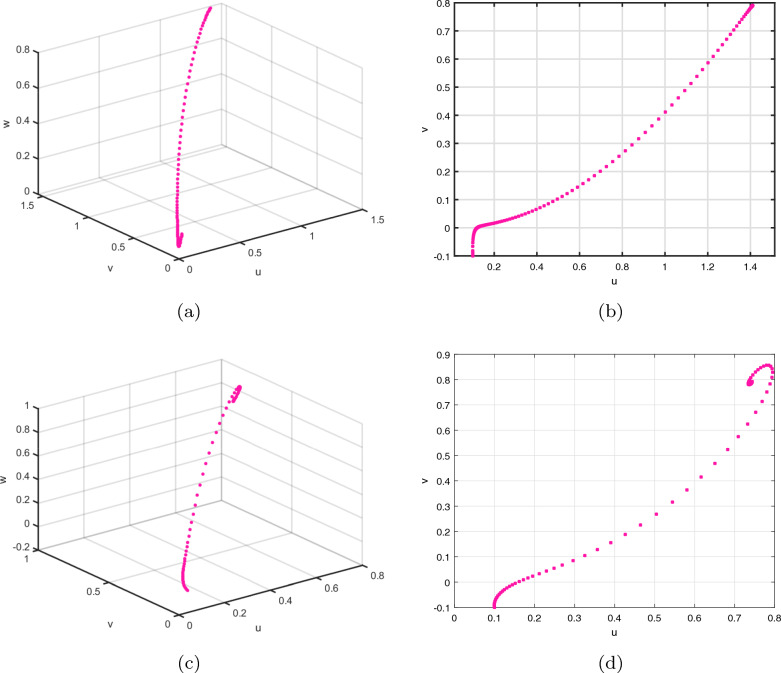
Attractors of the controlled (5.3) for $\delta =0.5$ and 0.97 with ICs $\big (\textbf{u}(0),\textbf{v}(0),\textbf{w}(0)\big )=(1.5,0.5,-0.5)$.

**Figure 13:**
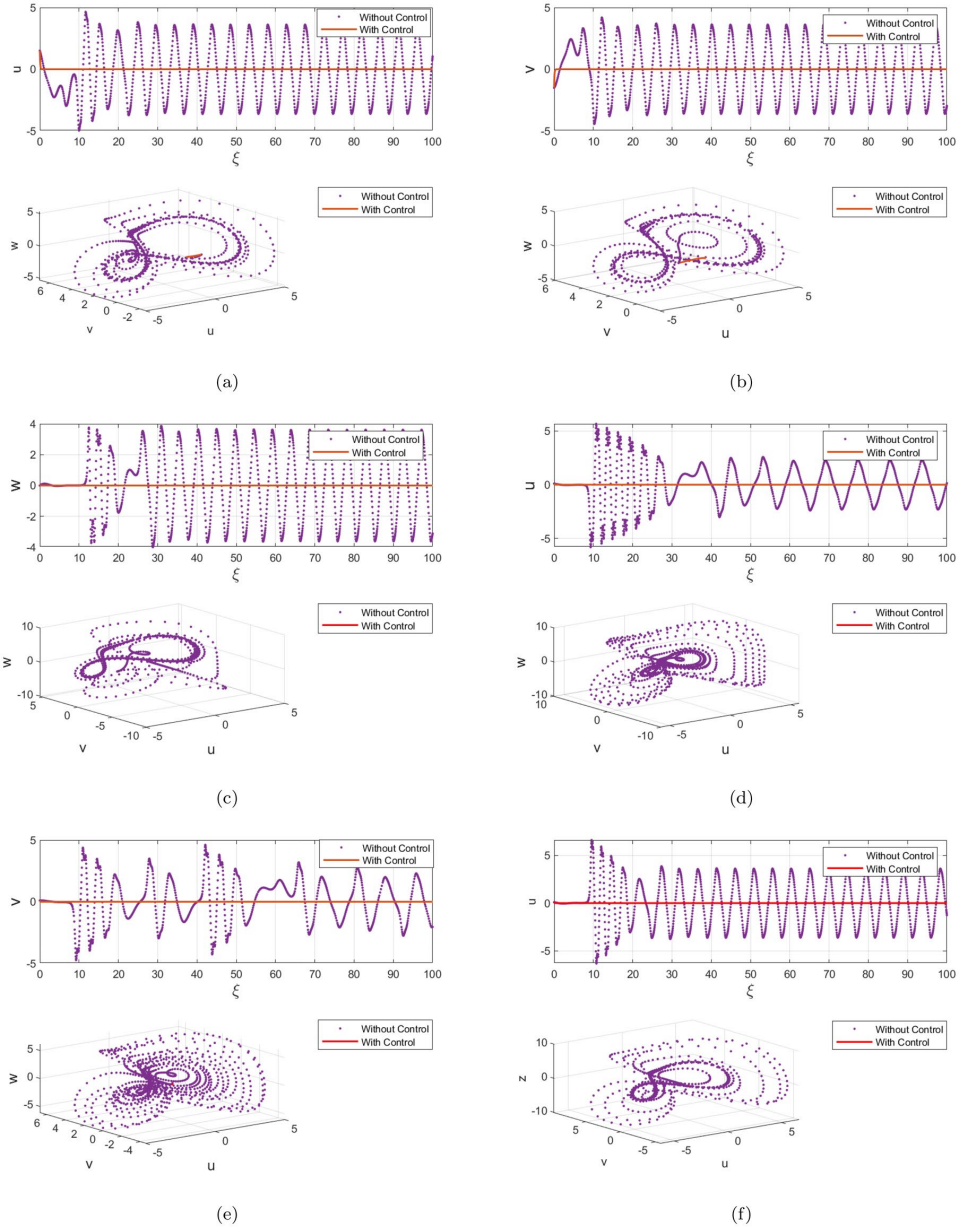
Chaotic attractor states of the controlled (5.3) having $\delta =0.5$ and $\delta =0.97$ with ICs $\big (\textbf{u}(0),\textbf{v}(0),\textbf{w}(0)\big )=(1.5,0.5,-0.5)$.

Furthermore, the computational modelling has been conducted in order to verify Theorem 5.2’s conclusions. Figures 12 and 13 show the time evolution of the satellite-based regulated fractional model (5.3). This validation of the DF-satellite model is essential to ensuring its reliability and stability. This diagnostic test and residual analysis can help identify any remaining instability or model misspecification. This can involve revisiting the data preprocessing steps, reevaluating the model selection and order, or exploring alternative modeling techniques. Iterative refinement can help improve the stability and accuracy of the fractional difference satellite model. The illustration clearly shows how the framework asserts near zero asynchronously, affirming its effective stabilization findings.

### Synchronization technique for fractional-order satellite model

Nonlinear regulators for coordinating the fractional-order satellite model are described in the subsequent section. The synchronization procedure seeks to minimize the difference between the master and slave visualizations, which compels it to gravitate towards zero. The master system is the commensurate fractional-order satellite model, represented by (2.14), while the slave system is characterized in the following manner:

5.7 $$\begin{aligned} {\left\{ \begin{array}{ll} \,^{c}\Delta _{\textbf{d}}^{\delta }{\textbf{u}}_{\textbf{n}}(\sigma ) =\frac{1}{3}{\textbf{v}}_{\textbf{n}}(\varsigma ){\textbf{w}}_{\textbf{n}} (\varsigma )-\textbf{d}{\textbf{u}}_{\textbf{n}}(\varsigma )+\frac{1}{\sqrt{6}} {\textbf{w}}_{\textbf{n}}(\varsigma )-{\textbf{u}}_{\textbf{n}}(\varsigma )+\mathbb {U}_{1}(\varsigma ),\\ \,^{c}\Delta _{\textbf{d}}^{\delta }{\textbf{v}}_{\textbf{n}}(\sigma ) =-{\textbf{u}}_{\textbf{n}}(\varsigma ){\textbf{w}}_{\textbf{n}}(\varsigma ) +b_{1}{\textbf{v}}_{\textbf{n}}(\varsigma )-{\textbf{v}}_{\textbf{n}}(\varsigma )+\mathbb {U}_{2}(\varsigma ),\\ \,^{c}\Delta _{\textbf{d}}^{\delta }{\textbf{w}}_{\textbf{n}}(\sigma ) = {\textbf{u}}_{\textbf{n}}(\varsigma ){\textbf{w}}_{\textbf{n}}(\varsigma )-\sqrt{6}{\textbf{u}}_{\textbf{n}}(\varsigma )-c_{1}{\textbf{w}}_{\textbf{n}}(\varsigma )+\mathbb {U}_{3}(\varsigma ),\end{array}\right. } \end{aligned}$$

where $\mathbb {U}_{1},~\mathbb {U}_2$ and $\mathbb {U}_{3}$ indicate the synchronization regulators.

The fractional error scheme is described as:

5.8 $$\begin{aligned} {\left\{ \begin{array}{ll} \,^{c}\Delta _{\textbf{d}}^{\delta }{\epsilon }_{1}(\sigma )=\Big (\frac{1}{3}{\textbf{v}}_{\textbf{n}} (\varsigma ){\textbf{w}}_{\textbf{n}}(\varsigma )-\textbf{d}{\textbf{u}}_{\textbf{n}}(\varsigma ) +\frac{1}{\sqrt{6}}{\textbf{w}}_{\textbf{n}}(\varsigma )+\mathbb {U}_{1}(\varsigma )\Big )\\ \qquad \qquad \qquad -\Big (\frac{1}{3}{\textbf{v}}(\varsigma ){\textbf{w}}(\varsigma )-\textbf{d}{\textbf{u}}(\varsigma ) +\frac{1}{\sqrt{6}}{\textbf{w}}(\varsigma )\Big )-\epsilon _{1}(\varsigma ),\\ \,^{c}\Delta _{\textbf{d}}^{\delta }{\epsilon _{2}}(\sigma )=\Big (-{\textbf{u}}_{\textbf{n}} (\varsigma ){\textbf{w}}_{\textbf{n}}(\varsigma )+b_{1}{\textbf{v}}_{\textbf{n}}(\varsigma ) +\mathbb {U}_{2}(\varsigma )\Big )\\ \qquad \qquad \qquad -\Big (-{\textbf{u}}(\varsigma ){\textbf{w}}(\varsigma ) +b_{1}{\textbf{v}}(\varsigma )\Big )-{\epsilon _{2}}(\varsigma ),\\ \,^{c}\Delta _{\textbf{d}}^{\delta }{\epsilon }_{3}(\sigma )= \Big ({\textbf{u}}_{\textbf{n}} (\varsigma ){\textbf{w}}_{\textbf{n}}(\varsigma )-\sqrt{6}{\textbf{u}}_{\textbf{n}}(\varsigma ) -c_{1}{\textbf{w}}_{\textbf{n}}(\varsigma )+\mathbb {U}_{3}(\varsigma )\Big )\\ \qquad \qquad \qquad -\Big ({\textbf{u}}(\varsigma ){\textbf{w}}(\varsigma )-\sqrt{6}{\textbf{u}}(\varsigma )-c_{1}{\textbf{w}} (\varsigma )\Big )-{\epsilon }_{3}(\varsigma ),\end{array}\right. } \end{aligned}$$

The suggested regulation govern that creates this synchronization system is described in the following theorem.

#### Theorem 5.3

Under the supposition of (5.7) and (5.8):

5.9 $$\begin{aligned} {\left\{ \begin{array}{ll} \mathbb {U}_{1}(\varsigma )=-\frac{1}{3}\big ({\textbf{v}}_{\textbf{n}}(\varsigma ) {\textbf{w}}_{\textbf{n}}(\varsigma )-{\textbf{v}}(\varsigma ){\textbf{w}}(\varsigma ) \big )+\textbf{d}\big ({\textbf{u}}_{\textbf{n}}(\varsigma )-{\textbf{u}}(\varsigma )\big ) -\frac{1}{\sqrt{6}}\big ({\textbf{w}}_{\textbf{n}}(\varsigma )-{\textbf{w}}(\varsigma )\big ),\\ \mathbb {U}_{2}(\varsigma )=\big ({\textbf{u}}_{\textbf{n}}(\varsigma ){\textbf{w}}_{\textbf{n}} (\varsigma )-{\textbf{u}}(\varsigma ){\textbf{w}}(\varsigma )\big )-b_{1}\big ({\textbf{v}}_{\textbf{n}} (\varsigma )-{\textbf{v}}(\varsigma )\big )-\delta _{2}\epsilon _{2}(\varsigma ),\\ \mathbb {U}_{3}(\varsigma )= -\big ({\textbf{u}}_{\textbf{n}}(\varsigma ){\textbf{w}}_{\textbf{n}} (\varsigma )-{\textbf{u}}(\varsigma ){\textbf{w}}(\varsigma )\big )+\sqrt{6}\big ({\textbf{u}}_{\textbf{n}} (\varsigma )-{\textbf{u}}(\varsigma )\big )+c_{1}\big ({\textbf{w}}_{\textbf{n}}(\varsigma )-{\textbf{w}} (\varsigma )\big )-\delta _{3}\epsilon _{3}(\varsigma ),\end{array}\right. } \end{aligned}$$

where $\delta _{2}\in (-1,2^{\delta }-1)$ and $\delta _{3}\in (0,2^{\delta }).$ Then, the master satellite model (2.14) and slave satellite model (5.7) are synchronized.

#### Proof

Plugging the regulate principle (5.11) in the fractional error system (5.8), we find:

5.10 $$\begin{aligned} \,^{c}\Delta _{\textbf{d}}^{\delta }\big (\epsilon _{1}(\sigma ),\epsilon _{2}(\sigma ), \epsilon _{3}(\sigma )\big )^{\textbf{T}}=\Phi \times \big (\epsilon _{1}(\sigma ), \epsilon _{2}(\sigma ),\epsilon _{3}(\sigma )\big )^{\textbf{T}}, \end{aligned}$$

where

5.11 $$\begin{aligned} \Phi =\begin{pmatrix} -(1+\chi _{1})&0&0\\ 0&-(1+\delta _{2})&0\\ 0&0&-\delta _{3} \end{pmatrix}. \end{aligned}$$

Since $\gamma _{1}=-(1+\chi _{1}),~\gamma _{2}=-(1+\delta _{2})$ and $\gamma _{3}=-\delta _{3}$ are the eigenvalues of (5.11). Thus, $\gamma _{\iota },~\iota =1,2,3$ comply with the stability the requirement mentioned in Theorem 5.1 for $\delta _{2}\in (-1,2^{\delta }-1)$ and $\delta _{3}\in (0,2^{\delta }),$ illustrating that the zero outcome of the fractional error model (5.7) is asymptotically stable, resulting in the synchronization of the master satellite model (2.14) and the slave satellite model (5.6). $\square$

**Figure 14:**
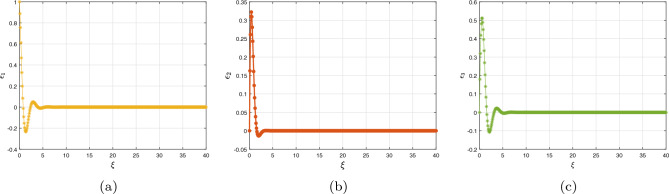
Error dynamics of (2.14) having $(\epsilon _{1},\epsilon _{2},\epsilon _{3})=(1.0,1.0,-1.0)$.

Mathematical computations using MATLAB are used to verify the truthfulness of this outcome. The parameters used are $\chi _{1}=0.4,~\chi _{2}=0.175,\chi _{3}=0.4$ and the initial settings are $(\epsilon _{1}(0),\epsilon _{2}(0),\epsilon _{3}(0))=(1.0,1.0,-1.0).$ Figure 14a–c depicts the time formation of the fractional error model’s contends (5.7). The graph unambiguously shows that erroneous values are often zero, affirming the efficacy of the previously addressed synchronization technique.

## Conclusion

This paper proposes a novel approach based on the commensurate and incommensurate fractional-orders DF-satellite and investigates its behavior for synchronizing chaotic attitude. The map’s assessment revealed a variety of unpredictable features, pointing out its dynamic diversity. The distinguished behaviours of the identified DF-satellite model have been studied for both COs and ICOs using various approaches to inspection including Lyapunov exponent computations, bifurcations and phase pictures. Furthermore, the system’s challenges have been determined employing the SpEn technique. The results highlight the significant impact of the network setting and fractional exponents on the configurations of the DF-satellite model. The numerical representations of such variables are crucial for influencing the structure and functioning of the framework, and fluctuations in their significance result in various paths as well as effects in the system’s state domain. Finally, the article suggests efficient oversight rules for ensuring the reliability and synchronization of the implemented system by manipulating its status to asynchronously tend to zero. The numerical analyses performed provide an extensive overview of the mechanism’s interactions and illustrate its fascinating and distinct behaviours, which have been crucial in investigating the consequences of fractional satellite models.

Simulation results confirm the robustness of the control methodology in chaos synchronization in the existence of different disrupting forces. Besides, internal disturbances (model uncertainties and parametric uncertainties) will be conducted in addition to external disturbances in the future work.

In upcoming studies, we will build the control mechanism for applying the discrete fractional form of Pyragas’ approach to the satellite system, utilizing a single delayed feedback variable. The benefit of Pyragas’ approach is that it requires no previous computations and has negligible real-time processing complexity. It is interesting to note that the angular velocity $\vartheta ,$ only the system state data employed in the control calculation-is all that is known. These studies indicate Pyragas’s discrete fractional calculus method will be the most remarkable research in control theory.

## Data Availability

The datasets used and/or analyzed during the current study available from the corresponding author on reasonable request.
